# The impact of economic freedom on COVID-19 pandemic control: the moderating role of equality

**DOI:** 10.1186/s12992-022-00800-0

**Published:** 2022-02-12

**Authors:** Guanglv Huang, Xiaoli Yu, Qinyi Long, Liqin Huang, Siyang Luo

**Affiliations:** Department of Psychology, Guangdong Provincial Key Laboratory of Social Cognitive Neuroscience and Mental Health, Guangdong Provincial Key Laboratory of Brain Function and Disease, Sun Yat-Sen University, Guangzhou, 510006 China

**Keywords:** COVID-19 pandemic control, Economic freedom, Equality

## Abstract

**Background:**

The absence of pharmaceutical interventions made it particularly difficult to mitigate the first outbreak of coronavirus disease 2019 (COVID-19). The current study investigated how economic freedom and equality influenced the pandemic control process.

**Methods:**

In Study 1, we assessed the effect of economic freedom and equality on COVID-19 pandemic control from nations worldwide. We collected the cumulative number of confirmed cases over time to perform logistic curve fitting and obtain the speed at which the first wave of the pandemic was controlled, and partial correlation analysis and representational similarity analysis (RSA) were performed to assess the similarity between economic freedom and the speed of pandemic control. In Study 2, an evolutionary game model in which economic freedom affects the speed of pandemic control through optimization of the allocation of available resources was developed. In Study 3, we used experimental manipulation to elucidate the psychological mechanism relating economic freedom and resource allocation.

**Results:**

The economic freedom of nation could be used to positively predict the speed of pandemic control and the related similarity pattern. Equality was found to moderate the correlation and representational similarity between economic freedom and the speed of pandemic control. The evolutionary game model revealed a mechanism whereby economic freedom influences the speed of pandemic control through high resource availability. Furthermore, cooperation was found to be a possible psychological mechanism explaining how economic freedom increases resource availability.

**Conclusions:**

Economic freedom has a positive effect on the control of the COVID-19 pandemic only among highly egalitarian nations. New interventions are needed to help countries heighten economic freedom and equality as they continue to battle COVID-19 and other collective threats.

**Supplementary Information:**

The online version contains supplementary material available at 10.1186/s12992-022-00800-0.

## Introduction

The COVID-19 pandemic caused by the novel severe acute respiratory syndrome coronavirus 2 (SARS-CoV-2) is still raging around the world. Although there have been transnational differences in the impact of COVID-19, the factors that cause such differences are poorly understood. In the face of this crisis that is almost without precedent in the human community, the basic state of society may be important in determining the size of its impact. Previous studies have found that psychosocial factors can affect the impact of COVID-19, such as individualism, tightness, and relational mobility [1–3]. However, there are still effects of many other potential social factors that have not been fully understood. The impact of COVID-19 differs between nations; some nations can control the pandemic more quickly. This shows that in addition to government policies, the social system of a country may also affect the speed of epidemic control, such as economic freedom. Szulczyk & Cheema found that countries with greater economic freedom had lower COVID-19 mortality rates [4]. Geloso and Murtazashvili argued that people in economically free societies will be less vulnerable during COVID-19 because they benefit from the wealth effect of freedom [5].

Economic freedom is defined as the freedom of the economy to not be subjected to government interference [6]. The core principle of economic freedom is the freedom of citizens to conduct economic activities with minimal interference from the state. The concept of economic freedom involves personal choice, voluntary exchange, freedom to enter markets and compete, and security of people and privately owned property [7]. Friedman [8] and Hayek [9] believed that a society with a high level of economic freedom could improve the effectiveness of the market in allocating resources. During a pandemic such as COVID-19, optimizing the deployment of resources is essential [10]. If this is the case, economic freedom may be a predictor of the success of anti-pandemic efforts. Few empirical studies have explored the relationship between economic freedom and pandemics. The study by Geloso & Bologna Pavlik revealed a buffering effect of economic freedom on gross domestic product (GDP) in the aftermath of the 1918 pandemic; specifically, they found that in countries with higher levels of economic freedom, the economy suffered less from the pandemic. Researchers argue that the process of resource reallocation to address the changed constraint of the pandemic is costly. For example, the cost of altering resource allocations is increased in the presence of burdensome regulations because they limit the ability of entrepreneurs to adjust. Therefore, freer economies are able to reallocate resources more quickly when facing a pandemic [11]. However, this study evaluated the impact of economic freedom on the economy, not the role of economic freedom on the development of the epidemic itself. As a characterization of the quality of a social system, economic freedom has a wide range of influences on many aspects of society. Stroup stated that a society with high economic freedom could improve the effectiveness of market resource allocation, thereby producing greater prosperity conditional on its resource endowments [12]. Thus, it can be posited that societies with high economic freedom possess great resource allocation capabilities and can prioritize the available resources for pandemic control, such as personal protective equipment and isolation treatment for infected people. Therefore, economic freedom may have a positive impact on COVID-19 pandemic control.

The fruits of economic growth are seldom distributed evenly among all economic groups, and inequality is real [13, 14]. It has been suggested that income inequality can be an indicator of a broader social context of structural inequality that affects health [15]. Equality is a key background driver of positive social phenomena. In the context of economic freedom influencing pandemic control, we expect a positive effect of economic freedom to appear only in societies with greater equality. The literature offers two main theories rationalizing the moderating effect of equality. The theory of innovation diffusion argues that advances in knowledge or technology that yield private benefits (such as advanced medical treatments) are initially primarily available to wealthy or highly educated people [16, 17]. When inequality is high, the diffusion of innovation from the rich to the poor may be slower. Neomaterial theory holds that inequality is related to the availability of resources [18]. An unequal society is imbalanced not only in the distribution of personal resources but also in the supply of infrastructure [19]. Historical research on smallpox revealed that if any subset of the population was excluded from access to flexible basic resources, such as food and medicine, the disease continued to spread and kill [20]. Based on these theories, we consider the role of economic freedom and equality in the context of COVID-19. We consider that under higher equality, the greater the economic freedom of a nation, the more effectively it can prioritize its limited resources for pandemic control and ensure that those resources are accessible to everyone, protecting society as a whole. However, in the case of lower equality and high economic freedom, the large amounts of available resources cannot be equally distributed to society. The bottom of society is deprived of access to resources, and the health of disadvantaged groups cannot be properly protected. Thus, the advantages of high economic freedom in pandemic control are obscured in more unequal societies. Schwartz’s egalitarianism is defined as a cultural value that emphasizes the transcendence of self-interest and the commitment to and support for the welfare of others [21]. Previous research has shown that cultures with more egalitarian values have a stronger association between intergroup contact and prejudice, which suggests that positive social contact can only be fully translated into good social attitudes in more egalitarian cultural contexts [22]. Schwartz’s egalitarianism is a subjective equality. The Gini coefficient is used to measure the income gaps among residents, which can be defined as an objective equality. In this study, we used Schwartz’s egalitarianism and the Gini coefficient as measures of equality.

In Study 1, we tested the effects of economic freedom and equality on COVID-19 pandemic control from nations worldwide, including the United States. Prior research has shown that the logistic regression model offers a good fit in explorations of the characteristics of the SARS pandemic [23]. For our study, we collected the cumulative number of confirmed cases over time to perform logistic curve fitting and obtain the speed at which the first wave of the pandemic was brought under control. After controlling for population density and underreporting of confirmed cases, we used partial correlation analysis to test the impact of economic freedom on the speed of pandemic control and representational similarity analysis (RSA) to test the similarity between economic freedom and the speed of pandemic control. RSA is a computational technique that uses pairwise comparisons of units to represent them in higher-order space [24]. We tested the moderating role of equality in the correlation and the representational similarity between economic freedom and the speed of pandemic control. To supplement the case for a causal interpretation of these results, Study 2 developed an evolutionary game model whereby economic freedom affects the speed of pandemic control through optimization of the allocation of available resources. Furthermore, in Study 3, we conducted a priming experiment to determine the psychological microfoundation of the mechanism connecting economic freedom and resource allocation.

### Study 1

In study 1, we collected data from 62 countries and 45 states in the United States to explore the relationship between economic freedom and epidemic control, as well as the moderating role of equality.

## Method

### COVID-19 pandemic control speed

The cumulative confirmed cases of COVID-19 in nations were obtained from the COVID-19 database of Johns Hopkins University [25]. The database was chosen because it has recorded all confirmed cases since the beginning of the COVID-19 outbreak. The data were obtained on August 4, 2020. We used the logistic function $\mathrm{y}=a/\left(1+{e}^{-k\left(x-{x}_c\right)}\right)$ to fit the cumulative confirmed cases over time. *x* is the number of days, *x*_*c*_ is the day when the curve rises fastest (the so-called turning point of pandemic control, after which the rise gradually slows), and y is the cumulative confirmed cases at *x* days. *a* is the maximum cumulative confirmed cases predicted by the function, and *k* is the growth rate of the cumulative confirmed cases curve, which denotes the overall speed of control of COVID-19. We adopted the same function for a total of 72 nations. Considering that as of August 4, 2020, multiple nations were facing a second wave of the pandemic and that the logistic function can measure curves with a single peak only, we set the cutoff date to exclude the second wave (see Supplementary materials). The start date is the day when the cumulative number reaches 100. Through logistic curve fitting, we obtained the overall control speed *k* and the day of the turning point *x*_*c*_.

Next, we set the period of pandemic control before *x*_*c*_ as stage 1 and the period after *x*_*c*_ as stage 2. To distinguish different control speeds at different stages, the function

$$ \mathrm{y}=\left\{\begin{array}{c}{a}_1/\left(1+{e}^{-{k}_1\left(x-{x}_{c1}\right)}\right),\kern0.5em 1\le \mathrm{x}<{x}_c\\ {}{a}_2/\left(1+{e}^{-{k}_2\left(x-{x}_{c2}\right)}\right),\kern0.5em \mathrm{x}>{x}_c\end{array}\right. $$

was used to refit the cumulative confirmed cases over time. *k*_1_ and *k*_2_ are the control speeds in stage 1 and stage 2, respectively. By counting the days before and after the turning point, we found that one nation had less than ten days before the turning point and nine nations had less than ten days after the turning point; these figures do not accurately reflect the early or late phase of pandemic control. Therefore, these nations were excluded. The analyses were thus conducted on the remaining 62 nations (Fig. S1). For the US states, we found that three had less than ten days before the turning point and two states had less than ten days after the turning point. The analyses were thus conducted on the remaining 45 states (Fig. S2).

### Economic freedom

The economic freedom index of nations in the world comes from the Fraser Institute’s “Economic Freedom of the World: 2019 Annual Report”, which measures the degree to which government institutions support economic freedom [7]. The index ranges from 0 (least free) to 10 (most free). The economic freedom index for the 50 US states comes from the Fraser Institute’s “Economic Freedom of North America 2019” [26]. It shows the extent to which each state government supports economic freedom and individuals are free from unfair economic restrictions. Both indexes range from 0 (least free) to 10 (most free). The degree of economic freedom is measured in five broad areas: Size of Government (e.g., Government consumption), Legal System and Property Rights (e.g., Judicial independence), Sound Money (e.g., Money growth), Freedom to Trade Internationally (e.g., Black-market exchange rates) and Regulation (e.g., Labor market regulations).

### Equality/egalitarianism of nations

Our equality measure is based on the cultural egalitarianism construct from the seven cultural value orientations proposed by Shalom Schwartz [27] and Gini coefficient as assessed by the World Bank [28]. Egalitarianism is defined as a cultural value that emphasizes the transcendence of self-interest and the commitment to and support for the welfare of others, which is a subjective variable. Egalitarianism is measured on a 9-point scale (− 1 to + 7). Respondents rate the importance of each item “as a guiding principle in MY life,” such as “as a guiding principle in MY life, the value of ‘equality’ is: contrary to my values (-1) to particularly important (+7).” Higher values indicate that nations attach more importance to interpersonal egalitarianism. This value was available for 50 of the 62 nations. The Gini coefficient was used to measure the income gap between residents and is an objective variable ranging from 1 to 0. The closer the Gini coefficient is to zero, the more equal the distribution of income is. The Gini coefficient was available for 56 of the 62 nations.

Racial inequality of states in the US comes from states’ poverty rate by race in 2019 [29]. The racial inequality of each state was calculated as follows: racial inequality = (black poverty rate - white poverty rate)/(black poverty rate + white poverty rate). The closer racial inequality is to 1, the more racially unequal the state is. The racial inequality measure was available for 40 of the 45 states.

### Population density

Population density is chosen as a covariate because it may promote more social contact, thereby increasing the chance of COVID-19 infection. The population density of nations is compiled from the United Nations Department of Economic and Social Affairs World Population Prospects 2019 [30]. and that of states comes from Statista [31], which uses population estimates in 2019 published by the US Census Bureau. Population density in each state is simply the population of a state divided by the area of the state.

### Underreporting index of COVID-19 confirmed cases

Following prior work, the underreporting index of cases is included as a covariate [3]. Russell et al. first calculated the corrected case fatality rate (CFR) for each nation, which was adjusted for the delay between admission to the hospital and death [32]. Then, they computed the ratio of the best empirical CFR estimate (1.4%) to the corrected CFR for each nation. If the ratio is smaller than 1, it means that the cases are underreported. Due to the different cutoff dates for the cumulative number of confirmed cases in each nation, we computed the corresponding underreporting index according to the specific cutoff date. This index is available for 61 of the 62 nations. Iceland had only 10 deaths in the first wave of COVID-19, so it was not included in the calculation of the underreporting index.

### Correlation analysis

Using the underreporting index and population density as covariates, Pearson’s partial correlation analysis between economic freedom and the overall control speed, stage 1 control speed, and stage 2 control speed was carried out to explore the impact of economic freedom on pandemic control speeds.

### Representational similarity analysis (RSA)

We conducted RSA by constructing similarity matrices for independent variables, dependent variables and covariates. Here, we take the operation of nations as an example. The steps to construct similarity matrices are as follows. First, 61*61 dissimilarity matrices were constructed for economic freedom, speeds of pandemic control, underreporting index and population density. The dissimilarity matrix refers to comparing 61 nations with each other and calculating the distance to form a diagonally symmetric dissimilarity matrix with zero values on the diagonal. Second, we subtracted each value of the dissimilarity matrix from the maximum value of the matrix and then divided the entire matrix by the maximum value to form a diagonally symmetrical similarity matrix with all values on the diagonal line being 1. Finally, each matrix was transformed into a vector of unique pairwise similarities by selecting values above the diagonal. Using the matrix vector of the underreporting index and that of population density as covariates, partial correlation was computed between the matrix vector of economic freedom and the matrix vector of control speed to obtain the representational similarity.

### Moderation analysis in correlation

In total, 50 nations have data on egalitarianism, and 56 nations have data on Gini coefficient. Pearson’s partial correlation coefficient between economic freedom and control speed of high egalitarianism/equality countries and low egalitarianism/equality countries was calculated respectively. The Fisher r-to-z transformation was used to assess the significance of the difference between two correlation coefficients. Additionally, within-person comparisons (via Steiger’s Z-Test) of correlations between economic freedom and stage 1 control speed and between economic freedom and stage 2 control speed were performed to determine whether economic freedom influenced pandemic control mainly in the early (and not late) stage [33].

### Moderation analysis in representational similarity

We computed RSA on economic freedom matrices and control speed matrices among highly egalitarian/equality nations (*r*_*high*_) and among less egalitarian/equality nations (*r*_*low*_), controlling for underreporting index matrices and population density matrices. Finally, significant tests were performed on the differences between the calculated similarities. For example, we tested whether egalitarianism/equality moderates the representational similarity between economic freedom and the overall control speed. Fisher z-transformation was used to convert *r*_*low*_ and *r*_*high*_ into *Z*_*low*_ and *Z*_*high*_ [34]. The formula

$$ {Z}_{low- high}=\left({Z}_{low}-{Z}_{high}\right)/\sqrt{1/\left({N}_{low}-3\right)+1/\left({N}_{high}-3\right)} $$

was used to test whether the difference between *r*_*low*_ and *r*_*high*_ reached a significant level.

A total of 40 states have data on economic freedom, racial inequality, and the speed of pandemic.

control. We classified states by the value of racial inequality, with low racial inequality states (*N* = 21) that are lower than the mean of racial inequality and high racial inequality states (*N* = 19) that are lower than the mean of racial inequality. The procedures of moderation analysis in RSA of data in states were similar to those of data in nations.

## Results

**Fig. 1:**
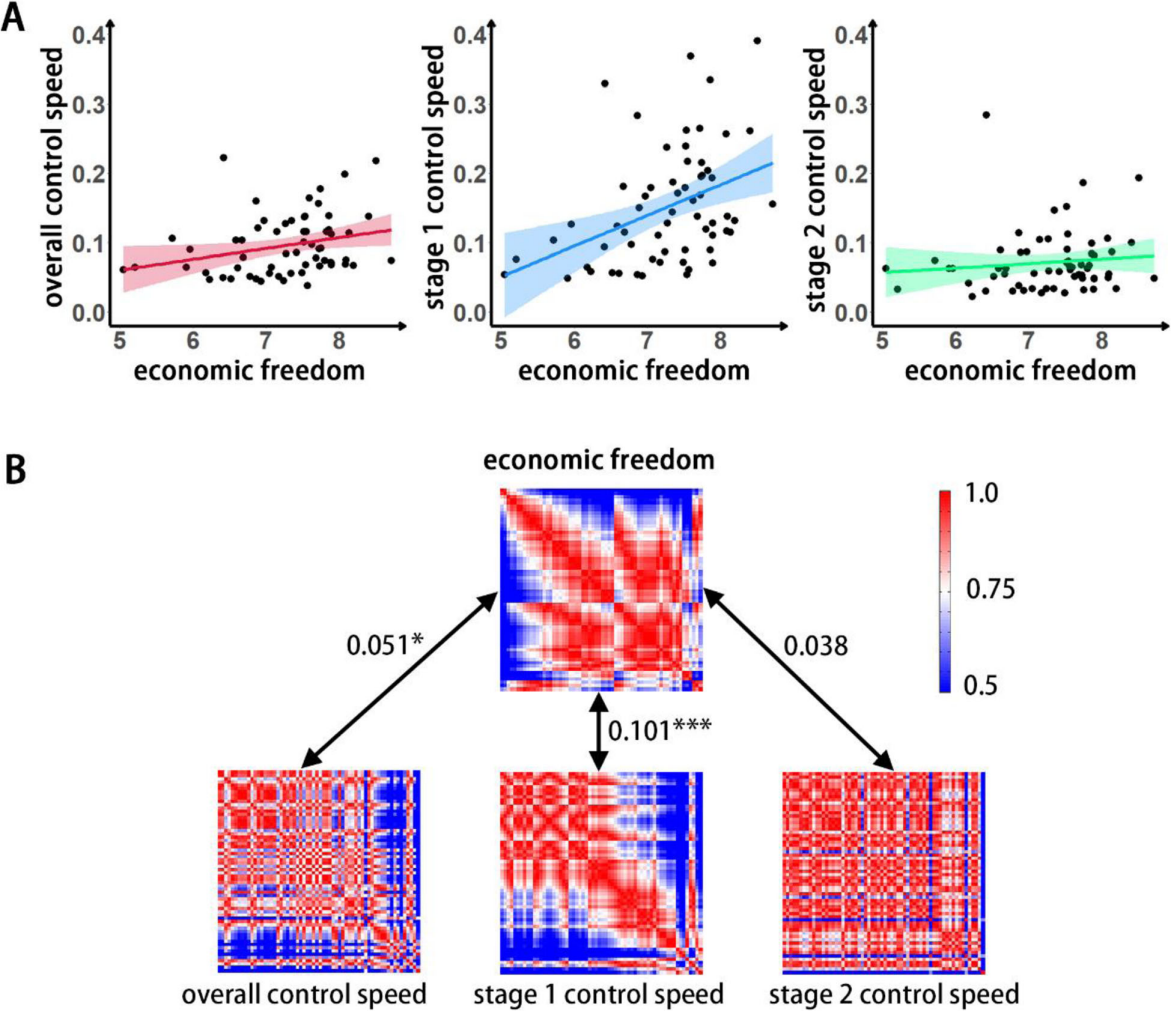
A) Correlation between nations’ economic freedom and the speed of pandemic control; B) RSA between the representation similarity matrix for nations’ economic freedom and that for the total controlling speed. The order of nations in the similarity matrix from top to bottom is Iraq, Iran, Ukraine, Pakistan, Bangladesh, Brazil, Kuwait, Saudi Arabia, South Africa, Greece, Morocco, Russia, Qatar, Mexico, Nigeria, United Arab Emirates, Poland, Slovenia, Hungary, Malaysia, Sweden, Bulgaria, Peru, Slovakia, Chile, Japan, Romania, Finland, Estonia, Denmark, Ireland, Canada, United States, United Kingdom, Singapore, Egypt, China, Thailand, Turkey, Serbia, Lebanon, Uruguay, France, Italy, Belgium, Norway, Lithuania, Germany, Czechia, Iceland, Latvia, Netherlands, Spain, Croatia, Portugal, Israel, Austria, Australia, Switzerland, Luxembourg, South Korea and New Zealand. * *p* < 0.05; **p < 0.01; *** *p* < 0.01

Partial correlation analysis revealed that economic freedom in 61 nations was positively associated with the overall control speed (*r* = 0.282, *p* = 0.030, Fig. 1). RSA revealed a significantly positive representation similarity between economic freedom and the overall control speed (*r* = 0.051, *p* = 0.030, Fig. 1), suggesting that two nations similar in levels of economic freedom are also similar in overall control speeds. Furthermore, economic freedom was found to be significantly associated with the stage 1 control speed but not the stage 2 control speed (stage 1, *r* = 0.407, *p* = 0.001; stage 2, *r* = 0.105, *p* = 0.429). To further examine whether the association between economic freedom and pandemic control is moderated by stage, we utilized Steiger’s Z-Test. The stage 1 control speed was found to be more strongly correlated (compared with the stage 2 control speed) with economic freedom (*z* = 2.549, *p* < 0.05). In addition, the similarity pattern of the stage 1 control speed was associated with the economic freedom similarity pattern (stage 1, *r* = 0.101, *p* < 0.001; stage 2, *r* = 0.038, *p* = 0.100), suggesting that two nations with a similar degree of economic freedom would be similar in the early stage of pandemic control but not the late stage.

**Fig. 2:**
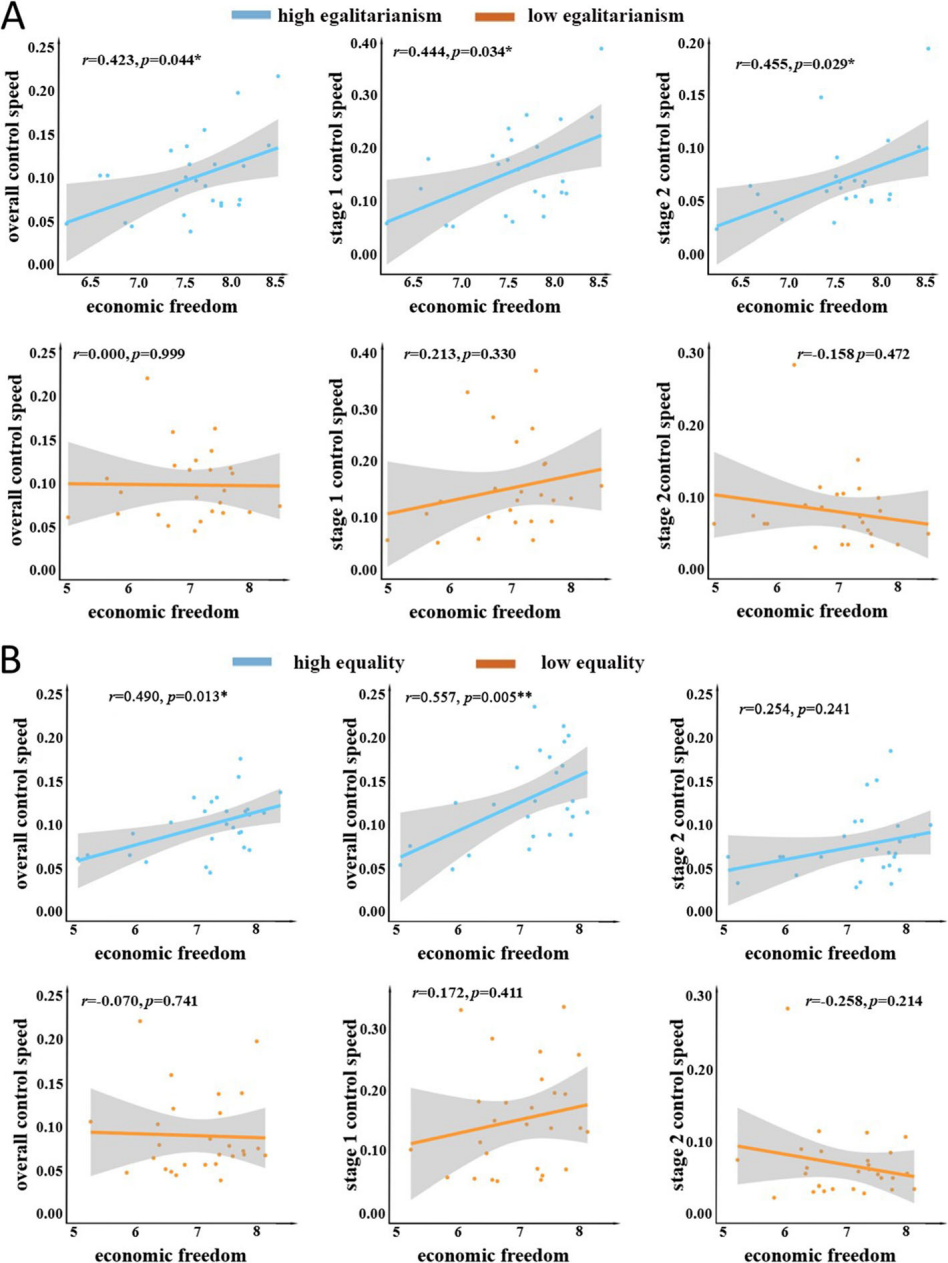
A) Correlation between high/low egalitarian nations’ economic freedom and the speeds of pandemic control. B) Correlation between high/low equal nations’ economic freedom and the speeds of pandemic control

The results further show that egalitarianism moderates the relationships between economic freedom and control speed (overall, *β* = 0.090, *p* = 0.027; stage 1, *β* = 0.152, *p* = 0.046; stage 2, *β* = 0.109, *p* = 0.011, Fig. 2A). A simple effect analysis reveals that economic freedom had a positive effect on control speeds only among highly egalitarian nations but not among nations with low egalitarianism (overall: *r*_*high*_ =0.423, *p* = 0.044; *r*_*low*_ =0.000, *p* = 0.999; stage 1: *r*_*high*_ =0.444, *p* = 0.034; *r*_*low*_ =0.213, *p* = 0.330; stage 2: *r*_*high*_ =0.455, *p* = 0.029; *r*_*low*_ = − 0.158, *p* = 0.472, Fig. 2A). Moderation analysis in RSA also suggested the moderating role of egalitarianism from a different angle. There is a positive similarity between the representational pattern of economic freedom and that of the overall control speed among highly egalitarian nations but not among less egalitarian nations (*r*_*high*_ =0.124, *p* = 0.032; *r*_*low*_ = − 0.062, *p* = 0.282, *Z*_*low* − *high*_ = − 2.28, *p* = 0.023). The moderation effect of egalitarianism on the stage 1 and stage 2 control speeds was similar to that on overall control speed (stage 1: *r*_*high*_ =0.187, *p* = 0.001; *r*_*low*_ = − 0.028, *p* = 0.632, *Z*_*low* − *high*_ = − 2.65, *p* = 0.008; stage 2: *r*_*high*_ =0.220, *p* < 0.001; *r*_*low*_ = − 0.036, *p =* 0.542; *Z*_*low* − *high*_ = − 3.16, *p* = 0.002, Fig. S3). To further verify the moderating effect of equality, we also used the Gini coefficient as an objective index of equality. The results showed that the Gini coefficient moderates the relationships between economic freedom and control speed (overall: *r*_*high*_ =0.490, *p* = 0.013; *r*_*low*_ = − 0.070, *p* = 0.741, *Z* = 2.10, *p* = 0.036; stage 1: *r*_*high*_ =0.557, *p* = 0.005; *r*_*low*_ =0.172, *p* = 0.411, *Z* = 1.58, *p* = 0.114; stage 2: *r*_*high*_ =0.254, *p* = 0.241; *r*_*low*_ = − 0.258, *p* = 0.214, *Z* = 1.81, *p* = 0.070, Fig. 2B, Fig. S4).

**Fig. 3:**
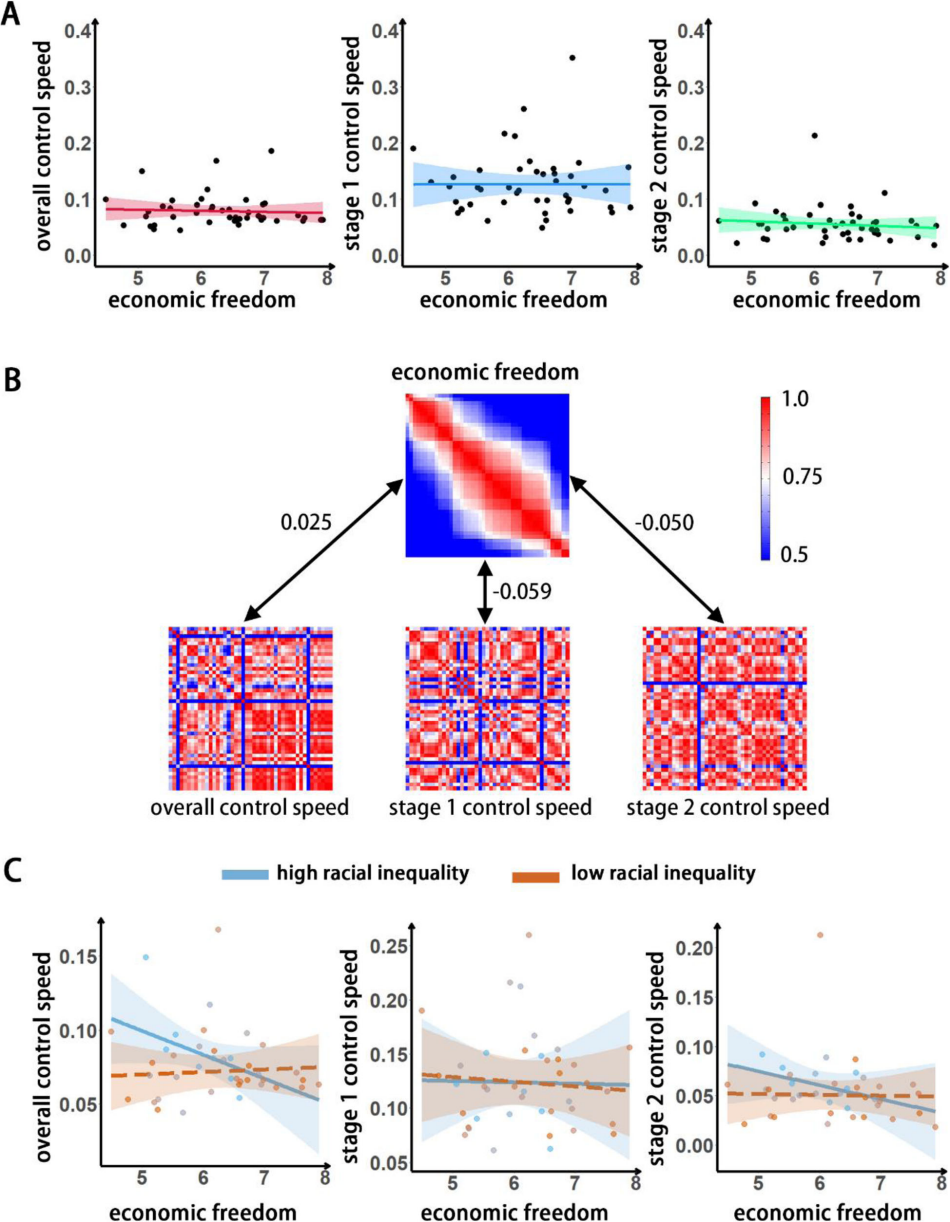
A) Correlation between states’ economic freedom with the speed of pandemic control; B) RSA between the representation similarity matrix for states’ economic freedom and that for the control speeds. C) Racial inequality moderates the correlation between states’ economic freedom and the speeds of pandemic control

Considering that the United States as a whole is a relatively unequal nation (listed as a nation with.

low egalitarianism and low equality), individual states’ degree of economic freedom may have no effect on pandemic control. The results confirmed no significant correlation between economic freedom and the overall control speed or stage 1 and stage 2 control speeds (overall, *r* = − 0.122, *p* = 0.434; stage 1, *r* = 0.056, *p* = 0.723; stage 2, *r* = − 0.146, *p* = 0.350, Fig. 3). RSA also revealed a nonsignificant result (overall, *r* = 0.025, *p* = 0.429; stage 1, *r* = − 0.059, *p* = 0.062; stage 2, *r* = − 0.050, *p* = 0.118, Fig. 3).

### Study 2

Through the analysis of public data, the first study revealed that economic freedom had a positive impact on the speed of epidemic control, and this impact was moderated by equality. However, the underlying mechanism of how economic freedom affects epidemic control could not be explored. To solve this problem, study 2 used agent-based modeling to construct an evolutionary game model of COVID-19. Evolutionary game models manipulate changes in economic freedom and equality to determine whether the speed of COVID-19 control will be affected.

## Method

### Model descriptions

We used NetLogo to simulate the impact of economic freedom and equality on COVID-19 pandemic control. The spread of COVID-19 among the population is considered to conform to the SEIRD model [35]. ‘S’ refers to the Susceptible, who is uninfected and may be infected after contact with an infected individual. ‘E’ refers to the Exposed, who has been infected with COVID-19 but has not become ill. ‘I’ refers to the Ill, showing symptoms. ‘R’ refers to the Recovered. People who have recovered will not be infected with COVID-19 again, nor will they be infectious. Finally, ‘D’ refers to the Dead. The different states of SEIRD are represented by different colors of the agent in the model. The transitions between states are some probability events. An agent representing a susceptible individual has three possible outcomes (not infected, recovered after infection, and dead) to describe the different results of individuals in the real world. Various agents move in a simulated environment. When the Susceptible has intensive contact with the Exposed or the Ill, the Susceptible may become the Exposed, thus entering the incubation period and beginning to have the ability to infect other Susceptible agents. After the incubation period, the Exposed enter the onset period and become the Ill. The onset period refers to the time from the onset of illness to recovery or death. The Ill may become the Recovered or the Dead as the onset period ends.

Constructing the SEIRD model of COVID-19, we considered the embodiment of economic freedom and equality in the model. According to the results of the aforementioned real-world study, economic freedom has a positive effect on the speed of COVID-19 pandemic control, and this positive effect depends on high equality. Given that different individuals in society have unequal possession of resources (such as wealth) and countries with high economic freedom can efficiently allocate resources during a crisis, we chose resources as the starting point for modeling. Resources are embodied as all attributes that help protect agents from harm. For example, agents with high resources tend to be less likely to be infected, enjoy more treatments after infection and are less likely to die. Before the pandemic, the initial resources occupied by individuals in society basically followed a normal distribution, showing a certain degree of gap between rich and poor. To distinguish countries with different resource equality, we choose to maintain the same mean of the normal distribution and change the standard deviation. The larger the standard deviation is, the more serious the gap between the rich and the poor in the country. The reason for controlling the same mean is that the goal of manipulation is the degree of equality of society rather than the degree of wealth. Economic freedom is reflected in the speed at which the total available resources are allocated to all agents during the simulation.

### Behavioral rules

Among the simulations of COVID-19, agents maintain their behaviors that are characterized by six simple rules and influenced by the whole interaction among them (see details in Supplementary materials). The six rules apply to all agents and happen in sequence (Figs. S5-S6).

### Rule one: moving

The rules of agents’ moving behavior patterns refer to the parameter settings of Cuevas [36]. Cuevas posited that individuals’ movement in the context of COVID-19 is a probabilistic behavior. The probability of staying in place should be greater than the probability of walking around, and the probability of moving short distances should be greater than the probability of moving long distances. The daily probability of movement for all agents obeys a uniform distribution: *P*_*Moving*_~U(0.2, 0.4).

### Rule two: infecting

In Gharakhanlou & Hooshangi’s spatiotemporal simulation for COVID-19 infection [37], they set the number of days of incubation period as a parameter that obeys a normal distribution: Incubation ∼ N(8, 2^2^) We follow their approach and give the same incubation period to the yellow agents who represented an infected state. The trigger condition for green agents to be infected by infected agents is close contact. Taking the position of a green agent as the center and within a circle with a radius of 1 unit, if there exists an infected agent, then the green agent will enter the judgment of whether it is infected. The probability of being infected obeys a normal distribution whose mean and standard deviation are related to CumuResource and Inequality, namely:

$$ \left\{\begin{array}{c}\mu =\left(-0.002\times \mathrm{CumuResource}+0.1\right)\times \left(-0.1\times \mathrm{Inequality}+1.5\right)+0.2\\ {}\sigma =\frac{\left(-0.002\times \mathrm{CumuResource}+0.1\right)\times \left(-0.1\times \mathrm{Inequality}+1.5\right)+0.2}{10}\\ {}{P}_{\mathrm{BeingInfected}}\sim \mathrm{N}\left(\mu, {\sigma}^2\right)\end{array}\right. $$

CumuResource influences the probability of infection, which suggests agents with high cumulative resources are less likely to be infected. Inequality changes the impact of CumuResource on the probability of being infected. *P*_BeingInfected_ in our simulation refers to the practice of Cuevas [36], which sets the probability of being infected in the range of 0.1 to 0.3. After being judged to be infected, a green agent will become a yellow agent representing an infected state and enter the incubation period.

### Rule three: being detected

Rule three simulates the process of detecting the infected agents, where detection is a probability event and will be affected by CumuResource. Each yellow agent may be detected every day in its incubation period. The detection formula is: *P*_*BeingDetected*_ = 0.00998 × CumuResource + 0.001 Once the detection occurs, the corresponding yellow agent will turn purple, which means that as a known infected agent, it will enjoy a slightly higher allocation proportion than the yellow agent in allocation of resources (see Rule six). The purple agents continue to pass the incubation period and infect green agents as yellow agents do.

### Rule four: falling ill

Khalili et al. conducted a meta-analysis of studies describing the epidemiological characteristics of COVID-19 published between December 1, 2019, and March 1, 2020 [38]. Rule four considers real-world statistics and uses them in our simulation. The yellow agents and the purple agents at the end of the incubation period will make a judgment to distinguish whether the final result is recovery or death. The formula to be executed is: *P*_*Die*_ =  − 0.0004 × CumuResource + 0.041. When a death event occurs, the yellow/purple agent turns red, which indicates that it is in the onset period, and its final result is death. The onset period of the red agents obeys the normal distribution, which is expressed by the formula: $\mathrm{Onset}\sim \mathrm{N}\left(0.02\times \mathrm{CumuResource}+15,{\left(\frac{0.02\times \mathrm{CumuResource}+15}{10}\right)}^2\right)$ This reflects that economically superior individuals in the real world can take on more forms of treatment when the condition is severe and reach the death result later. When a death event does not occur, the agent who has passed the incubation period will turn orange, which means that its final result is recovery. The onset period of the orange agents obeys the normal distribution as well:

$$ \mathrm{Onset}\sim \mathrm{N}\left(-0.02\times \mathrm{CumuResource}+19.5,{\left(\frac{-0.02\times \mathrm{CumuResource}+19.5}{10}\right)}^2\right) $$

Such setting reflects that individuals who are economically advantageous can enjoy more and better treatments and achieve faster recovery.

### Rule five: showing outcomes

The red and orange agents will enter different states after the onset period. The red agents will turn white, representing the state of death. The white agents will not perform the movement in Rule one or the allocation of resources in Rule six. The orange agents will finally become blue, a state denoting recovery. The blue agents represent individuals who have acquired antibodies after recovering from COVID-19; thus, they will no longer be infected.

### Rule six: allocating resources

A society with high economic freedom has high resource allocation capabilities and can provide a large amount of resources for supply in a short time [8, 9]. In this study, the economic freedom in the model is reflected in the speed of resource allocation under the condition that the total amount of resources remains equal. The purpose of controlling the total amount of resources is to distinguish between high and low economic freedom, rather than the large and small amount of overall resources. Economic freedom is expressed by the number of days allocated for resources (EconomicNotFreedom = {200,500}). A faster resource allocation speed means more available resources per day. Correspondingly, the average amount of resources that can be allocated per day is: $\mathrm{ResourcePerDay}=\frac{\mathrm{TotalResource}}{\mathrm{EconomicNotFreedom}}$ We then allocate resources to various agents based on two principles: the first is the principle of demand, and the second is the principle of inequality. Agents in different states have different demands for resources; thus, we use *r* to represent their allocation weight in the resource allocation process. The principle of inequality is represented by the Matthew effect. Wealth or resource advantages will be further strengthened; that is, the rich get richer, and the poor get poorer. Agents with higher initial resources will be allocated more resources each day. Therefore, we first calculate the relationship between the unit resources of total initial resources and the daily available resources and allocate resources to agents according to their initial resources and weights. The relationship is as follows:

$$ \mathrm{ResourcePerInitial}=\frac{\mathrm{ResourcePerDay}}{\sum {\mathrm{InitialResource}}_{<100}\times r} $$

The accumulated resources of an agent on day n + 1 is:

$$ {\mathrm{CumuResource}}_{n\kern0.5em +\kern0.5em 1}={\mathrm{CumuResource}}_n+\mathrm{ResourcePerInitial}\times \mathrm{InitialResource}\times \kern0.5em r $$

### Analytical techniques

Economic freedom was high when EconomicNotFreedom was set to 200, and economic freedom was low when EconomicNotFreedom was set to 500. Equality was high when Inequality was set to 5, and equality was low when Inequality was set to 10. This study focused on the speed of pandemic control. Consistent with the treatments in the real-world study, the overall control speed is obtained by fitting the cumulative number of infected agents over time. Then, we calculate the stage 1 control speed and the stage 2 control speed according to the turning point of pandemic control. We used interval estimation (95% confidence interval, 95% CI) of the dependent variable values under different conditions to reveal whether the effect existed. Nonoverlapping confidence intervals implies statistical significance (at the 0.05 level).

## Results

**Fig. 4:**
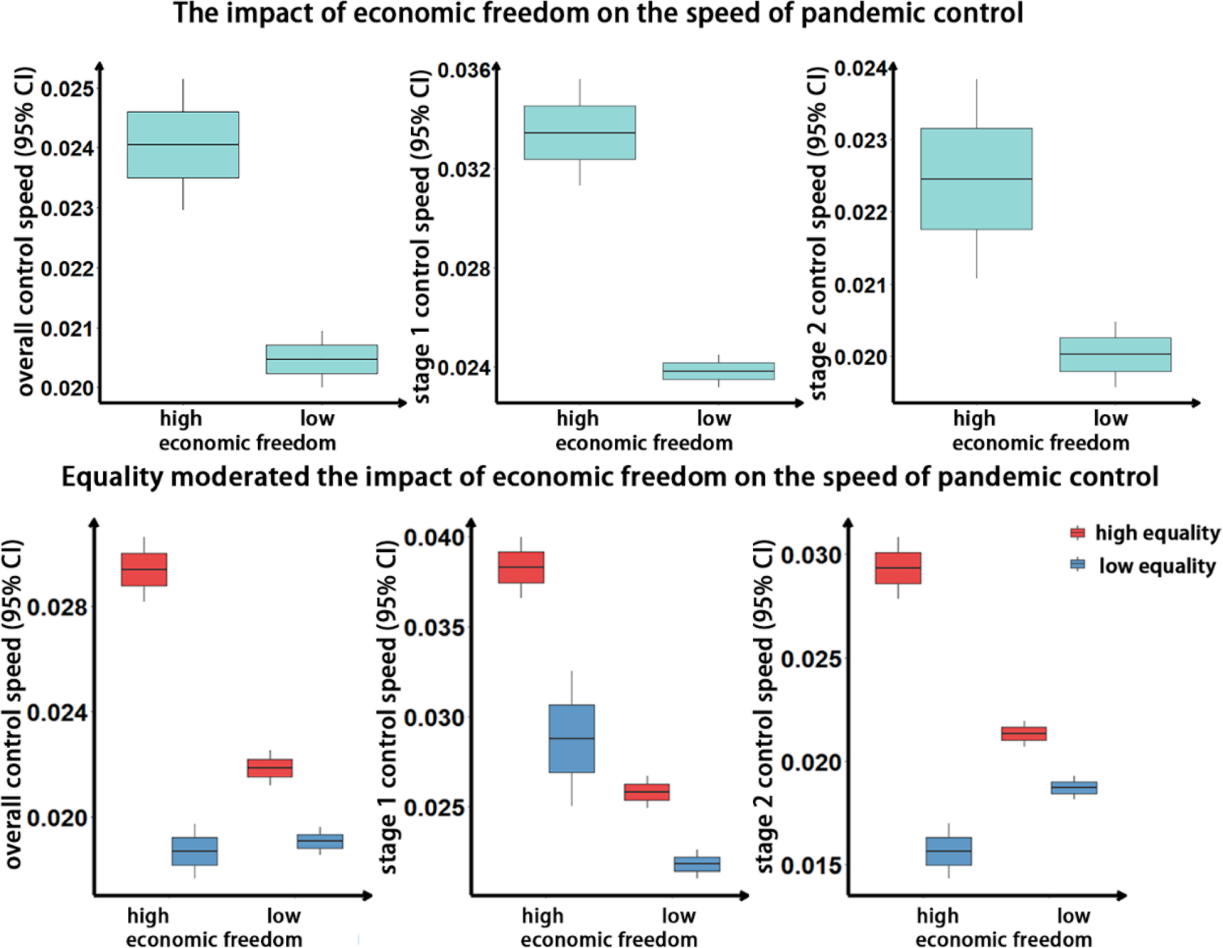
The impact of economic freedom on the speed of pandemic control. In the box plot, the box center was the mean; the box edges represented the standard error of the mean; and the box whiskers displayed the 95% confidence interval of the mean. The interpretation of the subsequent box plots was similar

The speeds of pandemic control under high economic freedom were higher than those under low economic freedom, and the corresponding 95% CIs did not coincide (overall: *M*_high _ free_ 95%CI = [0.024, 0.026], *M*_low _ free_ 95%CI = [0.020, 0.021]; stage 1: *M*_high _ free_ 95%CI = [0.031, 0.033], *M*_low _ free_ 95%CI = [0.023, 0.025]; stage 2: *M*_high _ free_ 95%CI = [0.022, 0.025], *M*_low _ free_ 95%CI = [0.019, 0.020], Fig. 4, Fig. S7). The results indicated that an increase in economic freedom (represented by resource allocation capacity) leads to an increase in the overall control speed, stage 1 control speed, and stage 2 control speed.

When equality was taken into consideration, the results showed that equality moderated the relationship between economic freedom and the overall control speed (see Fig. 4). An increase in economic freedom under the condition of high equality would lead to an increase in the overall control speed (*M*_high _ equaity & low _ free_ 95% CI = [0.021, 0.022], *M*_high _ equaity & high _ free_ 95% CI = [0.029, 0.032]), while an increase in economic freedom under low equality would not lead to a change (*M*_low _ equaity & low _ free_ 95% CI = [0.018, 0.020], *M*_low _ equaity & high _ free_ 95% CI = [0.018, 0.020]). In other words, only in the case of high equality could economic freedom positively affect the overall control speed. The differences in stage 1 control speed between high and low economic freedom increased as the equality increased (*M*_low _ equaity & low _ free_ 95% CI = [0.021, 0.024], *M*_low _ equaity & high _ free_ 95% CI = [0.025, 0.027], *M*_high _ equaity & low _ free_ 95% CI = [0.025, 0.026], *M*_high _ equaity & high _ free_ 95% CI = [0.036, 0.040]), which supported the moderating role of equality. The results of the stage 2 control speed were slightly different from those of the stage 1 control speed. When equality was high, economic freedom had a positive effect on stage 2 control speed (*M*_high _ equaity & low _ free_ 95% CI = [0.021, 0.022], *M*_high _ equaity & high _ free_ 95% CI = [0.029, 0.032]), but when equality was low, economic freedom played a negative role (*M*_low _ equaity & low _ free_ 95% CI = [0.018, 0.019], *M*_low _ equaity & high _ free_ 95% CI = [0.015, 0.017]), which indicated that equality moderated the relationship between economic freedom and stage 2 control speed. We found that equality moderates the effect of economic freedom in the model, supporting the moderating role of equality in Study 1.

### Study 3

We explored the relationship between economic freedom and epidemic control at the macro level in study 1. In study 2, we discussed the possible mechanism by which economic freedom influences epidemic control; for example, economic freedom may affect epidemic control through resource allocation. Therefore, in study 3, we performed empirical research to explore how economic freedom affects resource allocation at the individual level.

There is research demonstrating that individuals’ preference for economic freedom is significantly correlated with their prosocial behavior [39]. A previous study reveals that economic freedom will increase entrepreneurship [40], and entrepreneurs tend to have lower risk and loss aversion than employees [41]. Therefore, we used cooperation-led resources, risk aversion-led resources and loss aversion-led resources as the dependent variable.

### Study 3a

## Method

### Participants

We recruited 219 US participants on Amazon Mechanical Turk. Participants completed this experiment on the online survey platform Qualtrics. Fifteen participants were excluded because they filled in words that were not related to the requirements in the writing task. Therefore, the final sample included 204 participants (102 males, 102 females), and the age ranged from 21 to 60 years old (*M* = 37.85, *SD* = 10.46). Before data collection, the sample size required for the experiment was calculated through G*Power. The current sample size can detect a moderate main effect of economic freedom (f = 0.25, alpha = 0.05, power = 0.80). All participants obtained informed consent on the content of the experiment. Participants were first measured about the control variables of self-constructs, general distrust, tightness, just world, subjective socioeconomic status, and social value orientation. After that, they were involved in completing a writing task that primed them to different concepts of economic freedom. Finally, they took part in three resource tasks in the context of the pandemic.

### Experimental manipulation

To manipulate the concept of economic freedom, participants were randomly assigned to the economic freedom group (*N* = 70), the economic unfreedom group (*N* = 68), or the neutral group (*N* = 66). Participants in different priming groups read different materials, and the materials described the social information corresponding to the group. The economic freedom group reads an economically free society, the economic unfreedom group reads an economically unfree society, and the neutral group reads information about an ordinary society (see details in Supplementary materials). Use a question to check the manipulation of priming: “Living in such a society, to what extent do you feel that this society is economically free?”. A 7-point Likert scale was used, with 1 being “not at all” and 7 being “very strong”.

### Materials

### Cooperation-led resources

We used the pandemic public goods game to measure the cooperation of participants. Cooperation was reflected in participants’ preference for interpersonal interaction, and participants needed to weigh their own interests with those of others to make decisions. The pandemic public goods game was adapted from the classic paradigm of the public goods game. Participants were told that there were confirmed cases of COVID-19 around their community and that all 50 people in the community needed to purchase enough masks for daily protection. Each of them was given 100 US dollars and needed to complete the purchase in two ways. The first way was to buy masks by themselves. In this case, the unit price of masks was $5. Another way was to invest money in a crowdfunding project to buy masks collectively. In this case, the unit price of masks was $2, but the masks purchased collectively needed to be divided equally among all 50 people. Participants chose how much money they would invest in the crowdfunding project. The more money was invested in the crowdfunding project, the more willing participants were to contribute to the collective. The money that participants invested in the crowdfunding project represented their cooperation-led resources.

### Risk aversion-led resources

The risk aversion lottery game in the context of the pandemic was adapted from the lottery choice paradigm [32]. Risk aversion was manifested in the participants’ own preferences, and there was no conflict between their own interests and the interests of others. Participants were told that the nucleic acid test kits were helpful to help screen patients with confirmed COVID-19. Assume that the subject, as the person in charge of a company, needed to prepare enough kits for all employees of the company. Participants completed seven decisions to choose one from two options. One of the options was a safety option, which was “100% chance to get 100 kits”, and the other option was a risky option, from the higher expectation (50% chance to get 165 kits) to the lower expectation (50% chance of getting 3200 kits). We added up the expectations of each participant’s seven options, which were risk aversion-led resources.

### Loss aversion-led resources

The loss aversion lottery game in the context of the pandemic was adapted from the lottery choice task [33]. Like risk aversion, loss aversion was manifested in the participants’ own preferences. Suppose that the government introduced a series of economic stimulus plans during the COVID-19 pandemic. There was no loss in the Type A plan (100% probability of getting 100 USD), and the Type B plan may have a loss (such as 50% probability of losing 500 USD and 50% probability of winning 800 USD). Participants needed to make seven choices among the seven combinations of these two types of plans. In this study, the expectations of the seven options selected by the participants were summed, and the value obtained was the total amount of money invested by the participants, which denotes loss aversion-led resources.

## Results

**Fig. 5:**
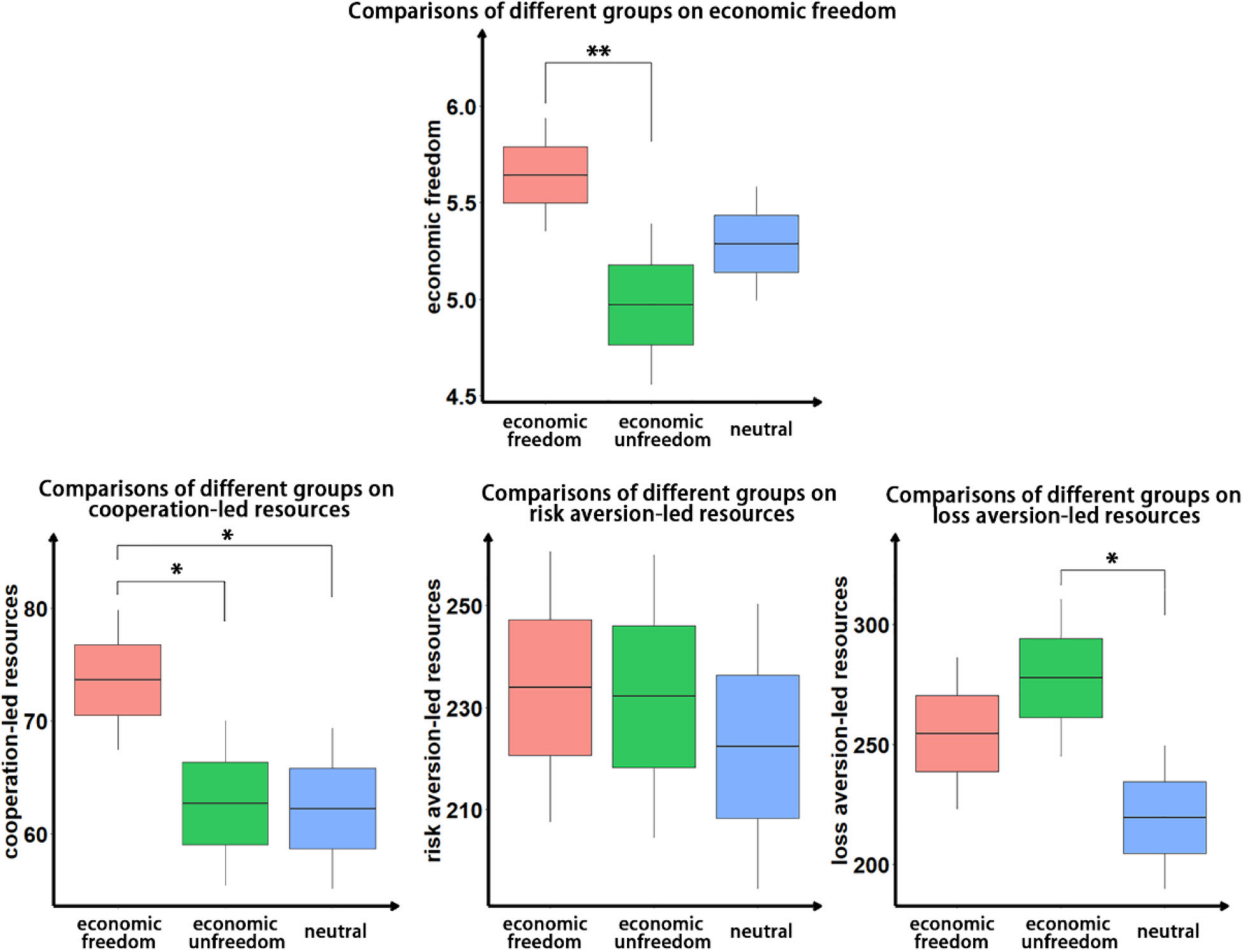
Comparisons of economic freedom, cooperation-led resources, risk aversion-led resources and loss aversion-led resources for different groups

The results show that compared with the economic unfreedom group and the neutral group, the economic freedom group reported higher economic freedom (*M*_free_ = 5.64, *SD* = 1.23; *M*_non − free_ = 4.97, *SD* = 1.72; *M*_neutral_ = 5.29, *SD* = 1.20, *F*(2, 201) = 3.97, *p* = 0.020, ${\upeta}_{\mathrm{p}}^2$ = 0.038, Fig. 5), which indicates that the priming of economic freedom is effective. Further post hoc (the false discovery rate method was used for multiple comparison correction) results show that the economic freedom group reported higher economic freedom than the economic unfreedom group (*M*_free_- *M*_non _ free_ = 0.67, FDR *p* = 0.015; *M*_free_- *M*_neutral_ = 0.35, FDR *p* = 0.192; *M*_non _ free_- *M*_neutral_ = − 0.32, FDR *p* = 0.192).

Subsequently, ANOVA was performed on cooperation-led resources, risk aversion-led resources, and loss aversion-led resources. Different priming groups had significant differences in cooperation-led resources (*M*_free_ = 73.64,*SD* = 26.11; *M*_non − free_ = 62.69,*SD* = 30.25; *M*_neutral_ = 62.21, *SD* = 29.08, *F*(2, 201) = 3.55, *p* = 0.031, ${\upeta}_{\mathrm{p}}^2$ = 0.034, Fig. 5). The resources of the economic freedom group were significantly higher than those of the economic unfreedom group and the neutral group (*M*_free_- *M*_non _ free_ = 10.95, FDR *p* = 0.038; *M*_free_- *M*_neutral_ = 11.43, FDR *p* = 0.038; *M*_non _ free_- *M*_neutral_ = 0.48, FDR *p* = 0.923), which indicates that participants who are primed with the concept of economic freedom express more cooperation in the pandemic public goods game. The results on risk aversion-led resources show that there was no significant difference (*M*_free_ = 234.00, *SD* = 111.22; *M*_non − free_ = 232.21, *SD* = 114.35; *M*_neutral_ = 222.38, *SD* = 113.64, *F*(2, 201) = 0.206, *p* = 0.814, ${\upeta}_{\mathrm{p}}^2$ = 0.002). Finally, there was a significant difference in loss aversion-led resources (*M*_free_ = 254.49, *SD* = 132.16; *M*_non − free_ = 277.63, *SD* = 135.56; *M*_neutral_ = 219.59, *SD* = 121.42, *F*(2, 201) = 3.380, *p* = 0.036, ${\upeta}_{\mathrm{p}}^2$ = 0.033). The resources of the economic unfreedom group were significantly higher than those of the neutral group, while there was no significant difference between the economic freedom group and the economic unfreedom group (*M*_free_- *M*_non _ free_ = − 23.14, FDR *p* = 0.297; *M*_free_- *M*_neutral_ = 34.90, FDR *p* = 0.179; *M*_non _ free_- *M*_neutral_ = 58.04, FDR *p* = 0.030). Combining the results of the three resource tasks, we found that cooperation can be a psychological mechanism that drives the effect of economic freedom on resource allocation efficiency.

### Study 3b

In study 3a, we discussed the influence of economic freedom on individual behaviors. The results show that economic freedom can promote individual cooperative behaviors. We added the equality variable in study 3b to explore whether equality moderates the effect of economic freedom on individual cooperative behaviors.

## Method

### Participants

We recruited 593 US participants from Amazon Mechanical Turk. The participants completed this experiment on the online survey platform Qualtrics. Sixty-two participants were excluded because they did not pass the forced choice or the time that they took to complete the questionnaire was greater than three standard deviations. Therefore, the final sample included 531 participants (310 males, 221 females) aged 18–64 years. All participants provided informed consent for the experiment. Participants first underwent an assessment of the control variables of self-constructs, general distrust, tightness, just world, subjective socioeconomic status, and social value orientation. Then, they completed a writing task that primed them to different concepts of economic freedom and equality. Finally, they participated in a cooperation-led resource task in the context of the pandemic.

### Experimental manipulation

To manipulate the concept of economic freedom and equality, participants were randomly assigned to the economic freedom-equality group (*N* = 132), the economic freedom-inequality group (*N* = 132), the economic unfreedom-equality group (*N* = 135), or the economic unfreedom-inequality group (*N* = 132). Participants in different priming groups read different materials, and the materials described the social information corresponding to the group. The economic freedom-equality group read about an economically free and equal society, the economic freedom-inequality group read about an economically free but unequal society, the economic unfreedom-equality group read about an economically unfree but equal society, and the economic unfreedom-inequality group read about an economically unfree and unequal society (see details in the Supplementary materials). The following two questions were posed to assess the manipulation effect of the priming: “Living in such a society, you feel that the society is economically free”, and “Living in such a society, you feel that the society is equal.” A 7-point Likert scale was used, with 1 being “Completely Disagree” and 7 being “Completely Agree”.

### Materials

#### Cooperation-led resources

These were the same as those in study 3a.

## Results

The results show that compared with the economic unfreedom group, the economic freedom group reported higher economic freedom (*M*_free_ = 5.76, *SD* = 1.02 ;*M*_non − free_ = 5.53, *SD* = 1.28, *t* = 2.292, *p* = 0.022), which indicates that the priming of economic freedom was effective. Compared with the unequal group, the equal group reported higher equality (*M*_equal_ = 5.79, *SD* = 1.00 ;*M*_inequal_ = 5.45, *SD* = 1.46, *t* = 3.166, *p* = 0.002), which indicates that the equality priming was effective.

**Fig. 6:**
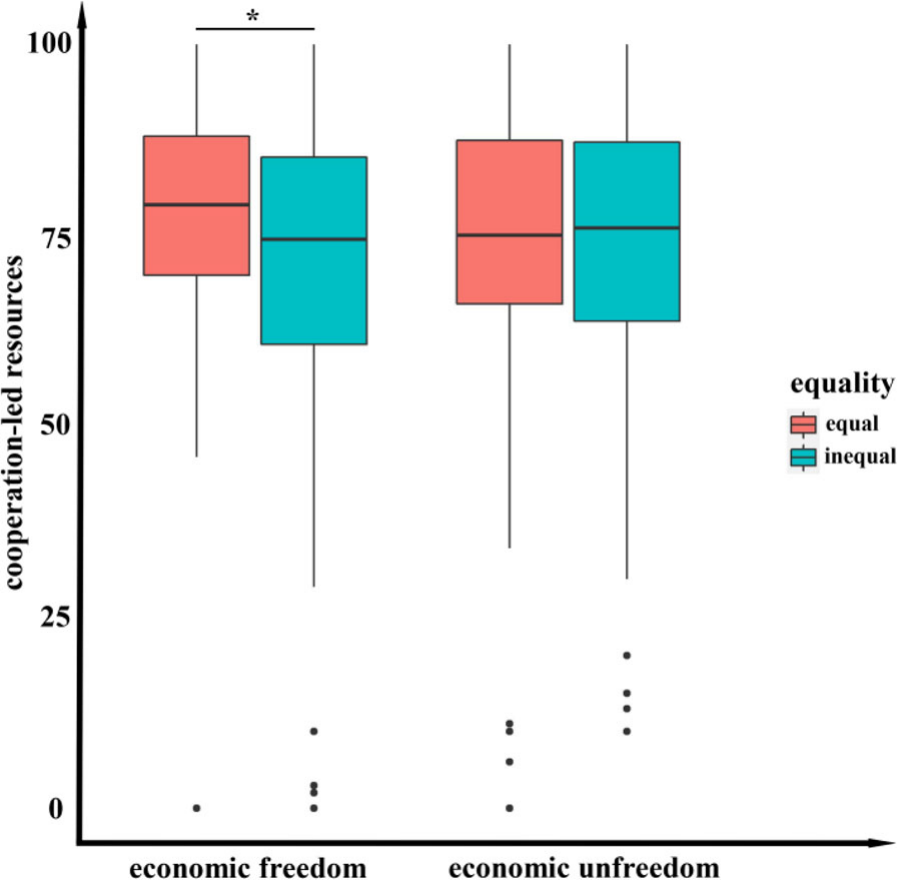
Comparisons of cooperation-led resources between scenarios with different equalities and economic freedoms

Subsequently, analysis of variance (ANOVA) was performed on cooperation-led resources, risk-aversion-led resources, and loss-aversion-led resources, where self-constructs, general distrust, tightness, just world, subjective socioeconomic status, and social value orientation were taken as the covariates. For cooperation-led resources, the main effect of economic freedom (*F* = 0.729, *p* = 0.393) and equality (*F* = 1.325, *p* = 0.250) was not significant, but the interaction was significant (*F* = 5.335, *p* = 0.021, Fig. 6). A simple effect analysis shows that the main effect of economic freedom was significant in the high-equality group (*M*_free_-*M*_non-free_ = 5.115, *p* = 0.025) and not significant in the low-equality group (*M*_free_-*M*_non-free_ = − 2.356, *p* = 0.305).

## Discussion

Research has found that the logistic function offers a good fit in analyses of the SARS pandemic [19]. Analyzing the COVID-19 pandemic, we found that the logistic function fit the cumulative confirmed cases of the first wave of COVID-19 well, which indicated that logistic function has applicability in describing infectious diseases. In contrast, Salvador et al. [3] performed a logarithmic transformation of the cumulative confirmed cases of the early stage, while Jiang et al. [1] calculated the cumulative confirmed cases within 30 days after the outbreak. The logic behind these treatments is that the number of infected individuals displays an approximately linear increase at the very beginning of a pandemic outbreak. As a consequence, these authors captured only the early spread of the pandemic, and the conclusions reached were limited to the beginning of the COVID-19 outbreak. However, we overcame this limitation to a certain extent by using logistic fitting not only to measure the overall control speed of the first wave of COVID-19 but also to distinguish two specific stages of pandemic control to discuss the factors affecting the control speed. Therefore, our conclusions about the role of economic freedom in COVID-19 control are applicable to a wider time frame.

There have been few studies on economic freedom and pandemics. However, existing studies have shown that economic freedom can reduce damage from pandemics. Countries with higher economic freedom were less affected by the 1918 pandemic and could restore economic order more quickly [11]. Some researchers have also explored the role of economic freedom in the context of the COVID-19 pandemic. For example, Chen et al. showed no connection between economic freedom and the number of deaths caused by COVID-19 [42]. Szulczyk & Cheema found that countries with greater economic freedom had lower COVID-19 mortality rates [4]. Rather than focusing on deaths, we posited that economic freedom, as an indicator of the quality of social systems, could impact society’s efforts to control the spread of COVID-19. Therefore, economic freedom might affect not only COVID-19 deaths but also pandemic control. The results based on public data and evolutionary game models showed that economic freedom had a positive impact on pandemic control and that this impact was achieved through the efficient allocation of resources. In addition, economic freedom was found to be significantly associated with the stage 1 control speed but not the stage 2 control speed. This result is due to the moderating effect of equality. Early-stage pandemic control is represented by the slowing growth of confirmed cases, while late-stage control is manifested by curbing the increase until the spread is stopped. The former could be achieved by individuals taking personal protective measures. However, the latter requires more powerful public measures, such as issuing lockdown orders and social distancing. Therefore, the level of government intervention increases in stage 2. For the high egalitarian societies, but not low egalitarian societies, the relationship between economic freedom and pandemic control remained positive for both stage 1 and stage 2. At stage 2, the government began to intervene, thus affecting the role of economic freedom. We considered that with the deep involvement of the government, highly egalitarian countries with high economic freedom are able to allocate resources fairly and quickly, thus affecting the speed of epidemic control. However, in low egalitarian nations, the government distributes resources unequally in stage 2 and allocates more resources to higher social classes. Furthermore, the rapid resource allocation capacity related to economic freedom does not apply to all citizens, so economic freedom has no effect on the speed of epidemic control in low egalitarian nations. Therefore, in stage 2, after summing the main economic freedom effects of high egalitarian countries and low egalitarian countries, the overall main effect was found to be insignificant. Nonetheless, we expanded the literature on the role of economic freedom in the field of infectious diseases. Together with research on tightness [2], individualism [1], and relational mobility [3], our findings proved that social factors do have an impact on COVID-19 control.

We also explored the impact of economic freedom on individuals. The concept of economic freedom originally characterized the social context, with little attention given to how economic freedom affects individual behavior. McCannon connected participants’ perception of economic freedom with their performance in economic games and concluded that economically free individuals participate more in wealth-creating investments [39]. However, as McCannon explained, the study, as a correlation exploration, could not prove that economic freedom indeed affects the economic behavior of individuals. Based on this work, we primed individuals with different concepts of economic freedom and equality. In study 3a, we assessed the influence of economic freedom on individual behavior. The results show that economic freedom can promote individual cooperative behavior. In study 3b, we added the variable of equality to further explore the moderating role of equality in economic freedom and individual behavior. We found that individuals primed with the concept of economic freedom showed higher cooperation in the pandemic public goods game in the high equality group. However, in the low equality group, economic freedom had no effect on individual cooperative behavior. Briefly, this study explored the possibility that economic freedom may influence individuals’ cooperative behavior and, thus, further influence the speed of social resource allocation. A previous study revealed that economic freedom promotes trust [43]. Therefore, we suspect that economic freedom may promote individuals’ trust in others, which makes individuals more willing to cooperate with others. Further research showed that in an equal society, high economic freedom endows individuals with a greater tendency to cooperate. It is possible that individuals in highly economically free societies are more likely to trust others and trust the government to distribute resources fairly. However, in an unequal society, the government cannot allocate resources fairly. Therefore, economic freedom may not promote trust, leading to the failure of economic freedom to affect the cooperative behavior of individuals. In a society with high equality and high economic freedom, individuals are more willing to cooperate and make contributions to the collective; thus, more resources are available in a highly economically free society. More available resources can be used to better protect individuals from infection. Even if individuals become infected, they can be isolated and treated in a timely manner to prevent the infection from spreading, which ultimately promotes faster pandemic control in societies with economic freedom.

Both neomaterial theory [16] and innovation diffusion theory [18] emphasize resource availability. Deep-rooted structural inequality hinders the resource access of disadvantaged groups in society. The prerequisite for controlling COVID-19 is to ensure that all individuals have access to adequate resources. If new resources are disproportionately allocated to the upper class of society, their distribution from top to bottom will be quite slow, leaving some people unable to enjoy the benefits of high economic freedom. The consequence of restricted use of resources for some populations is that infectious diseases continue to spread, causing pandemic control measures to remain ineffective [20]. The negative consequences of inequality in the context of COVID-19 support Mackenbach’s view that inequality is harmful to all members of society and that reducing inequality can safeguard everyone’s interests [44]. The combination of high economic freedom and high equality guarantees that society as a whole can properly protect itself in a pandemic.

This article used multiple methods to comprehensively explore the impact of economic freedom and equality on the speed of pandemic control, elucidating the underlying mechanism. Based on our findings, there are some problems that could be explored in future research. First, we analyzed pandemic control for the first wave of COVID-19 only. However, many nations have experienced multiple waves of COVID-19. Since vaccine production remains on the agenda and economic freedom should influence the distribution of vaccines, we believe that economic freedom might also play a positive role in the next few waves of COVID-19. Second, a society with high economic freedom has many advantages. Our model simulated only high resource allocation ability and abstracted from other mechanisms. Finally, the results of study 3 showed that priming individuals’ economic freedom would promote better cooperation. The results inspire us to explore whether individual cooperative behavior can affect resource allocation in the market and, thus, affect epidemic control, which needs to be confirmed by further research. Although the current results can clarify how economic freedom affects pandemic control, future research can explore other possible paths.

## Conclusions

The process of globalization generally unfolds together with the increasing degree of economic freedom in countries. The current studies demonstrated that economic freedom has a positive effect on the control of the COVID-19 pandemic only among highly egalitarian nations. Poor economic freedom and social inequality can make it take longer to control the COVID-19 pandemic or even perpetuate it. Therefore, new interventions are needed to help countries heighten economic freedom and equality as they continue to battle COVID-19 and other collective threats.

## Supplementary Information

**Additional file 1.** Supplementary materials.

## Data Availability

The data used in this study were obtained from the COVID-19 database of Johns Hopkins University (https://github.com/CSSEGISandData/COVID-19); the Economic Freedom of the World: 2019 Annual Report (https://www.fraserinstitute.org/studies/economic-freedom-of-the-world-2019-annual-report); The Kaiser Family Foundation State Health Facts (https://www.kff.org/other/ state-indicator/poverty-rate-by-raceethnicity/); and Statista (https://www.statista.com/statistics/183588/population-density-in-the-federal-states-of-the-us/). Other data are available from the corresponding author on request.
