# Supplementary material for: The impact of economic freedom on COVID-19 pandemic control: the moderating role of equality
Source: Global Health. 2022 Feb 12;18:15. doi: 10.1186/s12992-022-00800-0 (PMC8841047; doi:10.1186/s12992-022-00800-0)
Supplement: Supplementary file 1 — Additional file 1. Supplementary materials. [file 12992_2022_800_MOESM1_ESM.docx]

**Supplementary materials for**

**The Impact of Economic Freedom on COVID-19 Pandemic Control:**

**The Moderating Role of Equality**

**This PDF file includes:**

Supplementary modeling note

Supplementary methods & results

Figures S1-S6

**Study 1**

**Results**

**Figure S1. Fitting diagrams of the first wave of the COVID-19 pandemic in nations by stage**

The black line indicates the cumulative daily confirmed cases between day 100 and the data collection deadline. The blue line represents stage 1, and the red line represents stage 2.


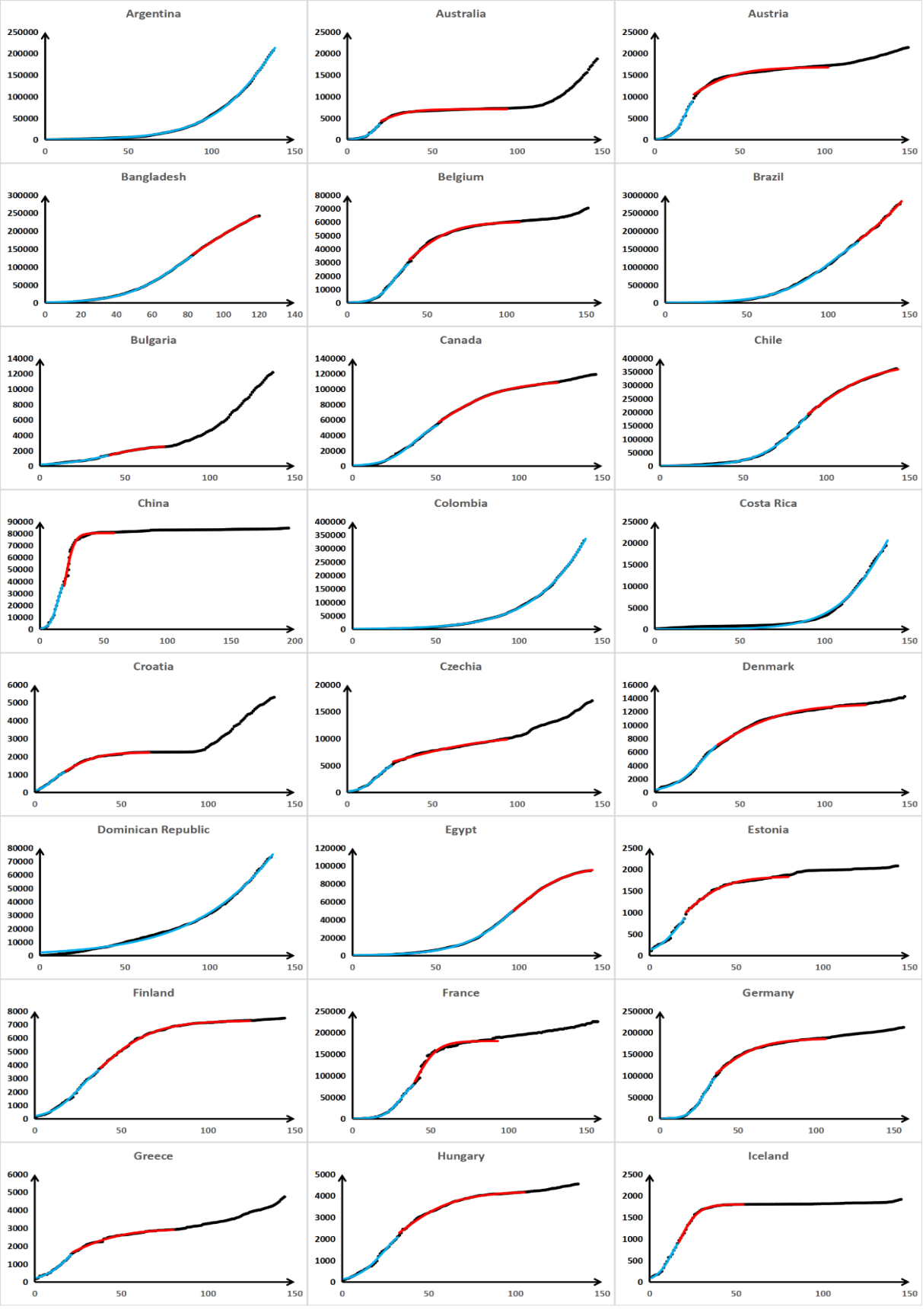


**
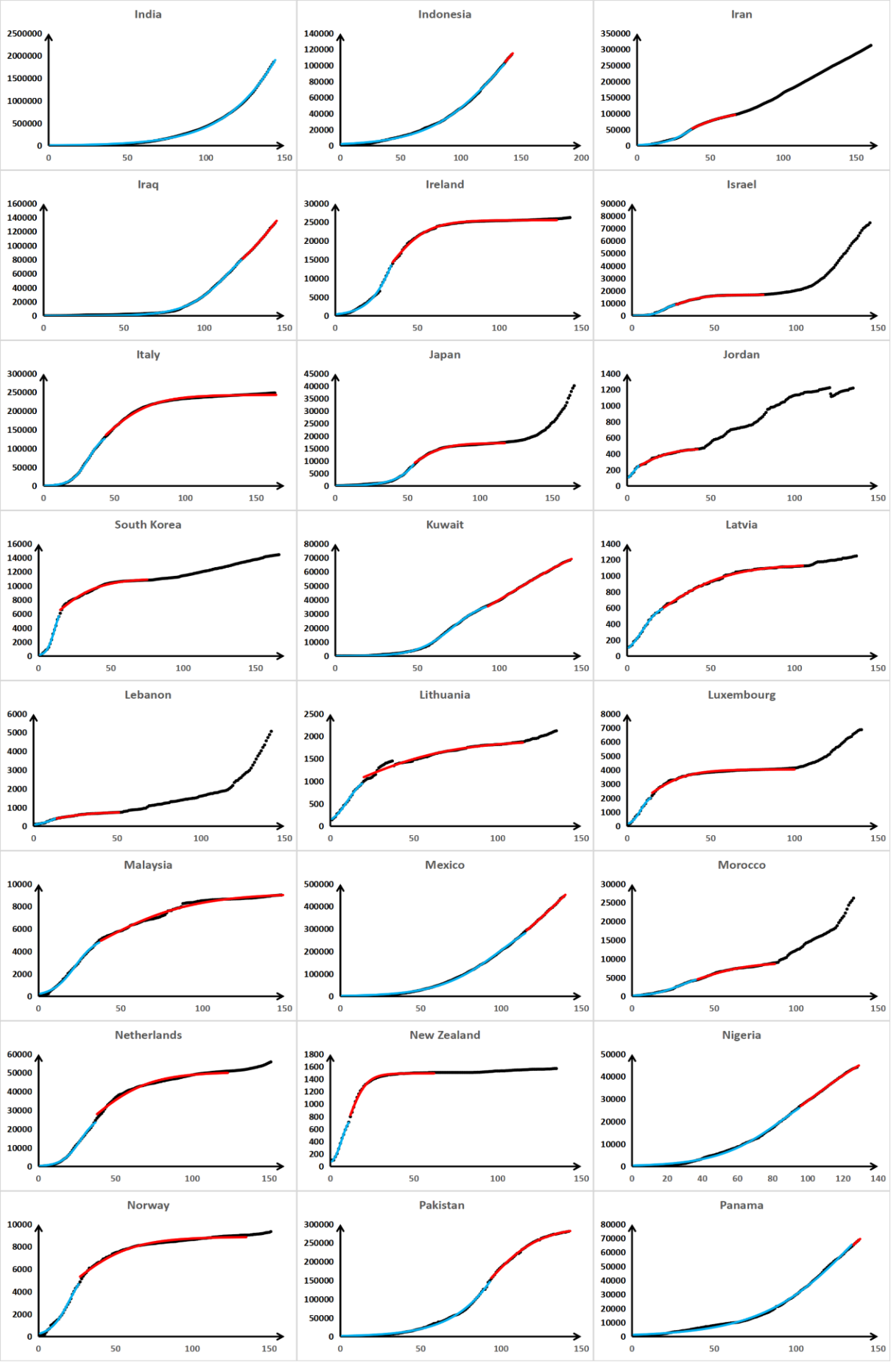
**

**
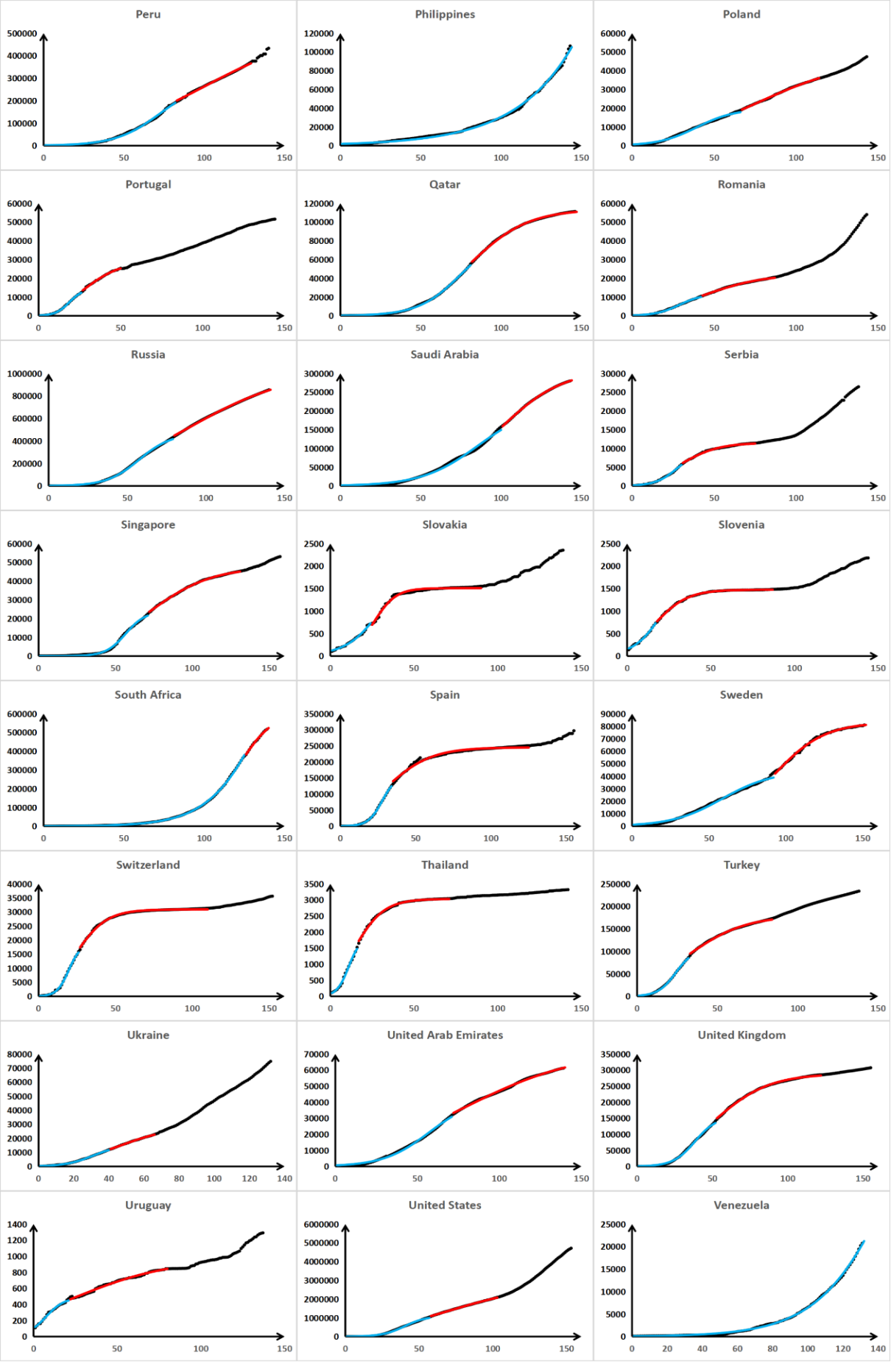
**

Figure S2. Fitting diagrams of the first wave of COVID-19 pandemic in states by stages

**
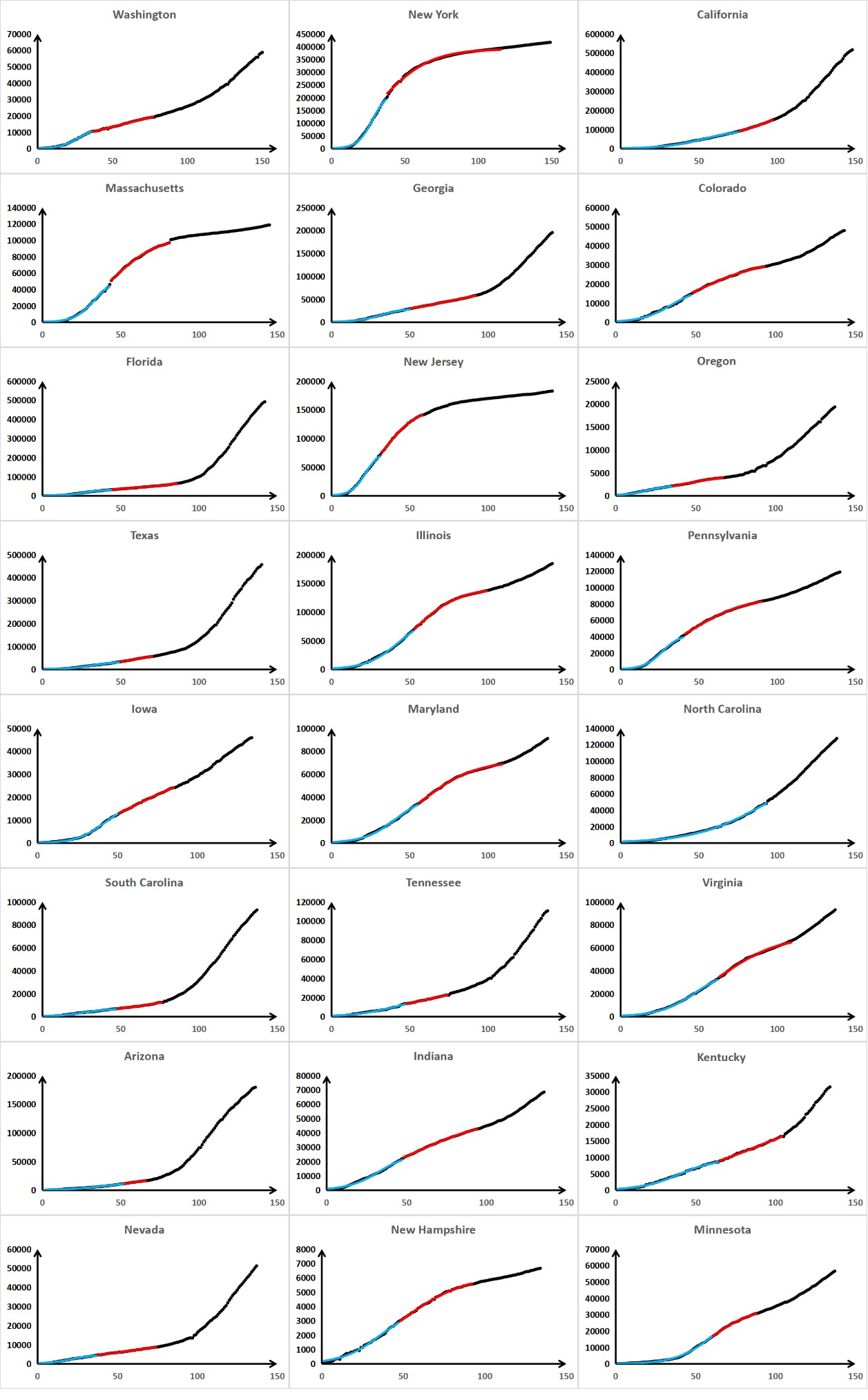
**

**
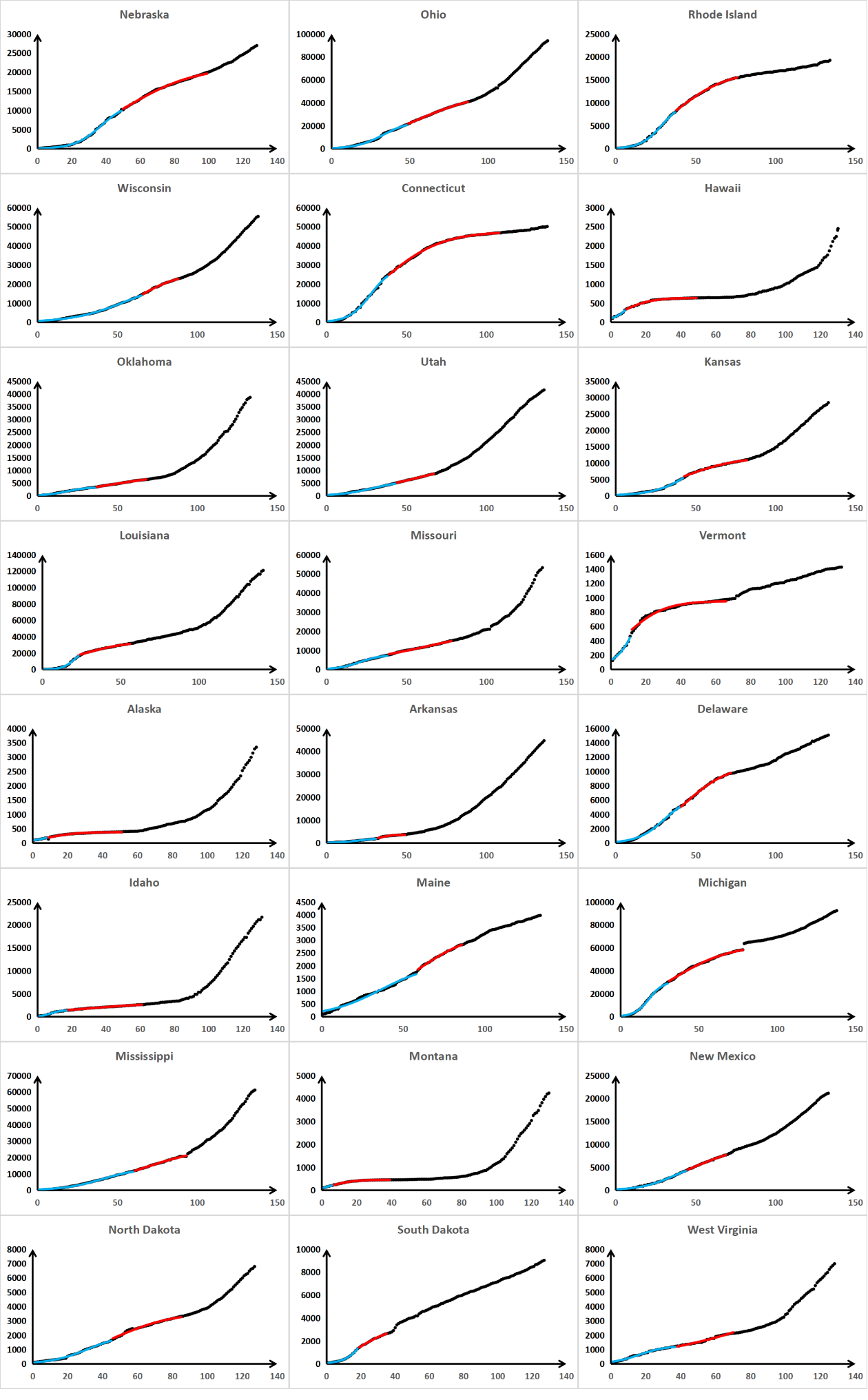

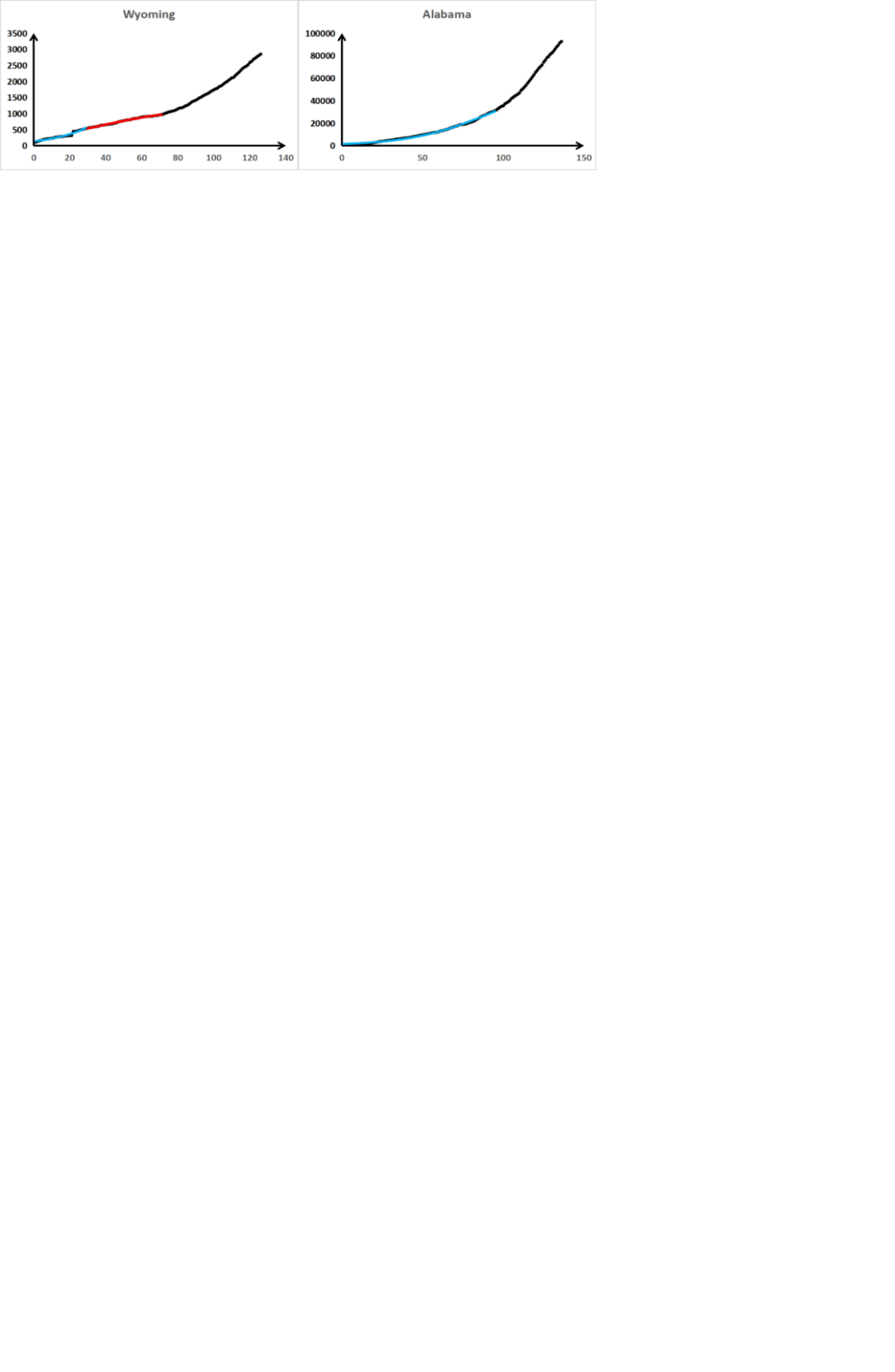
**

**
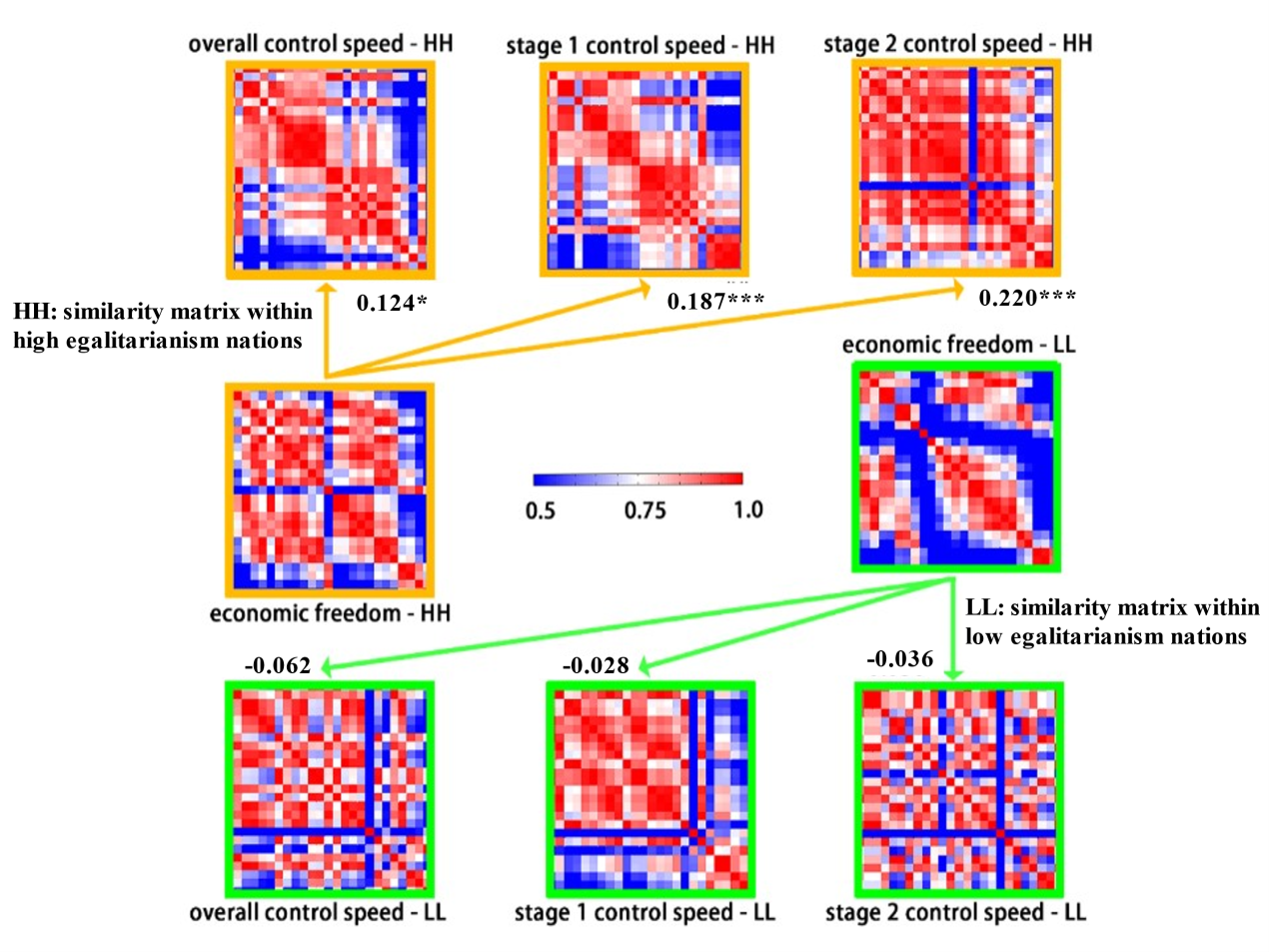
Figure S3.** Egalitarianism moderates the representational similarity between economic freedom and the speed of pandemic control of the nations. The green frames are the similarity matrices of low-egalitarianism nations, and the orange frames are the similarity matrices of high-egalitarianism nations.

**
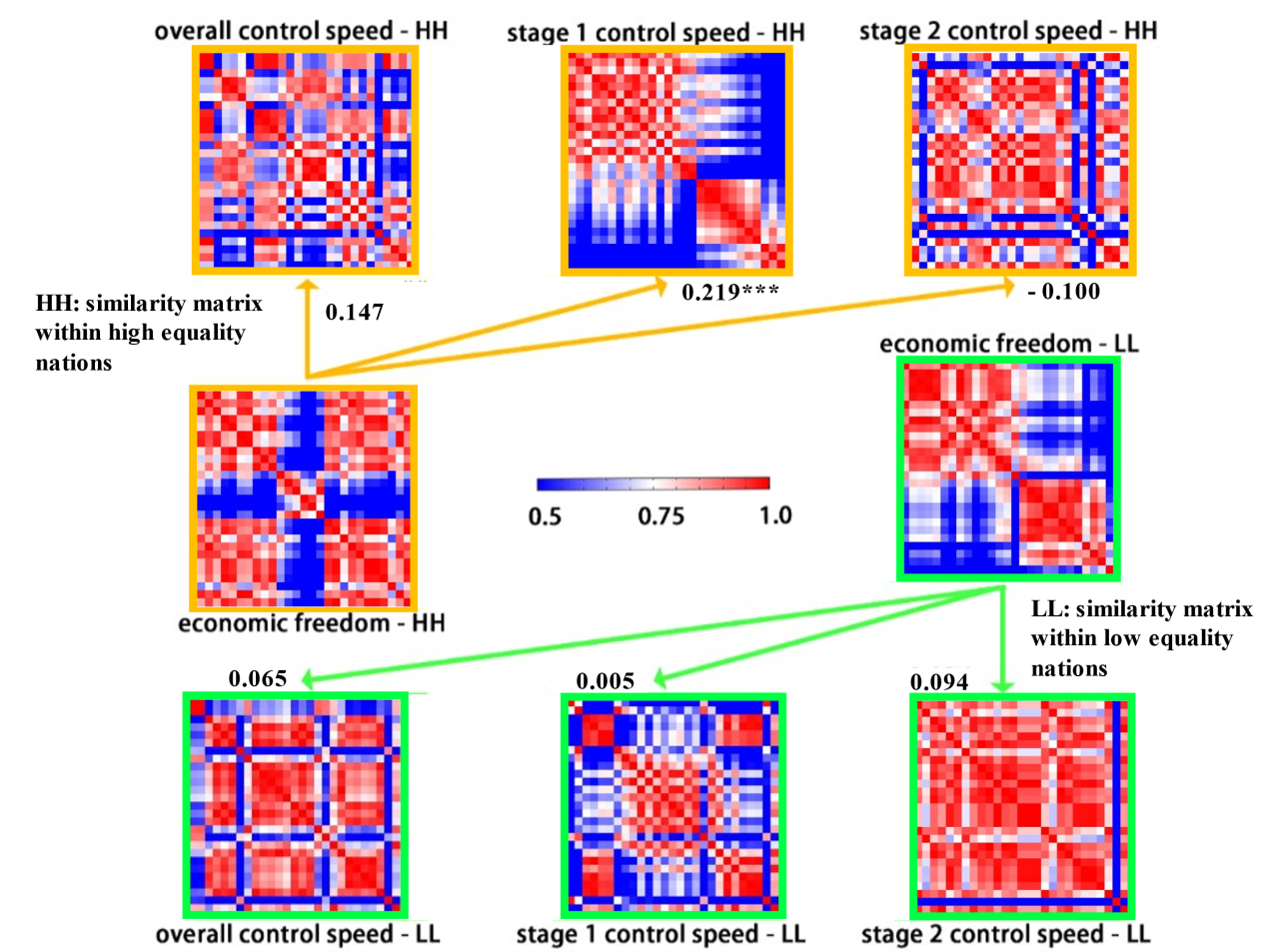
Figure S4.** Equality moderates the representational similarity between nations’ economic freedom and the speed of pandemic control. The green frames are the similarity matrices of low-equality nations, and the orange frames are the similarity matrices of high-equality nations.

The regression analysis results show that when per capita GDP and economic freedom were used as independent variables, neither could significantly predict the epidemic control speed, and economic freedom had a larger effect size than GDP. Representational similarity analysis (RSA) reveals a significant positive representation similarity between economic freedom and epidemic control speed (overall, r=0.044, p=0.063; stage 1, r=0.064, p=0.006; stage 2, r=0.054, p=0.021) when per capita GDP, population density and underreporting estimates were taken as the control variables, which suggests that two nations with similar levels of economic freedom have similar epidemic control speeds.

The economic freedom index of nations in the world comes from the Fraser Institute’s “Economic Freedom of the World: 2019 Annual Report”, which measures the degree to which government institutions support economic freedom. The degree of economic freedom is measured in five broad areas: Size of Government (e.g., Government consumption), Legal System and Property Rights (e.g., Judicial independence), Sound Money (e.g., Money growth), Freedom to Trade Internationally (e.g., Black-market exchange rates) and Regulation (e.g., Labor market regulations). Gross domestic product (GDP) is the total monetary or market value of all finished goods and services produced within a country’s borders in a specific time period. Previous studies have shown a significant correlation between GDP and economic freedom, and countries with high economic freedom generally have higher GDP.1 However, GDP and economic freedom have different priorities. GDP is concerned with national economic growth. The core principle of economic freedom is the freedom of citizens to conduct economic activities with minimal interference from the state, which reflects the economic flexibility of the country. The regression analysis results indicate that the impact of economic freedom on the speed of epidemic control is not determined by per capita GDP. Higher-dimensional RSA results show that two nations with similar levels of economic freedom have similar epidemic control speeds after taking the per capita GDP as a control variable. These results demonstrate the role of economic freedom in epidemic control.

**Study 2**

**Methods**

**Initialization**

The model environment is represented by a coordinate axis. The intersection of the horizontal axis and the vertical axis is zero (0,0). The minimum ($L_{x}$) and maximum values ($U_{x}$) of the horizontal axis constitute the range of the agents’ lateral movement. Similarly, the minimum ($L_{y}$) and maximum values ($L_{y}$) of the vertical axis constitute the range of the agents’ longitudinal movement. In this study, the size of the model environment is set to 300*300 ($L_{x}=-150$, $U_{x}=150$, $L_{y}=-150$, $U_{x}=150$), which means that the motion space of agents in the behavior interaction process is 90,000 units. There are a total of 9000 agents in the set model, 10 of which are marked in yellow, representing individuals who have been exposed to COVID-19, and the remaining 8990 agents are indicated in green, representing healthy and uninfected individuals. The initial positions of the yellow agents are at zero, while those of the green agents are random, indicating that the distribution of uninfected agents in the environment is random. All agents will be given resources. The distribution of initial resources in the populations adheres to a normal distribution with a mean of 30 and a standard deviation of inequality:

$$InitialResource\sim N(30,\mathrm{Inequality}^{2})$$

Inequality is a parameter used to indicate whether the distribution of initial resources is equal. It has 2 values ($Inequality=\{5,10\}$). The larger the value is, the larger the simulated gap between the rich and poor in society, and the greater the inequality between people. We set the resources of an agent during the simulation at no less than 0 or greater than 100. Therefore, an initial resource less than 0 will be set to 0, and an initial resource greater than 100 will be set to 100. In addition, each agent is given a parameter (CumuResource) describing the accumulated resources. The accumulated resources on day 1 are equal to the initial resources. Since agents will participate in the allocation of resources (see Rule 6), the accumulated resources of agents will continue to increase during the simulation. When CumuResource is not less than 100, the agent will stop participating in resource allocation to satisfy the condition that the accumulated resource of every agent falls within the range of 0 to 100, as follows:

$$CumuResource\in\left[ 0,100 \right]$$

**Behavioral rules**

**Rule 1: Moving**

Agents judged not to move on day n+1 will maintain their position on day n. Once an agent is determined to move, the probability of moving a short distance is 0.8, and the probability of moving a long distance is 0.2. The post-movement position on day n+1 of agents judged to be moving a short distance is calculated based on the position on day n, namely:

$${{x_{n+1}=x}_{n}+rand(-1,1)\times2 \atop{y_{n+1}=y}_{n}+rand(-1,1)\times2}$$

Function $rand(-1,1)$ extracts a value from the range of -1 to 1. The purpose of short-distance movement is to make the agents’ horizontal and vertical coordinates change by no more than 2 units based on the previous position. If an agent is judged to be moving a long distance, its position on day n+1 is:

$${{x_{n+1}=L}_{x}+rand\left( 0,1 \right)\times\left( {U_{x}-L}_{x} \right) \atop{y_{n+1}=L}_{y}+rand\left( 0,1 \right)\times\left( {U_{y}-L}_{y} \right)}$$

The long-distance movement formula randomly generates a new position in the global scope of the environment.

**Rule 2: Infecting**

The extent of the decrease in $P_{\mathrm{BeingInfected}}$ caused by the increase in CumuResource under a low-inequality condition is larger than that under a high-inequality condition. Due to

$$CumuResource\in\left[ 0,100 \right]$$

when $I\mathrm{nequality}=5$, $P_{\mathrm{BeingInfected}}$ of an agent with 0 CumuResource is

$$P_{\mathrm{BeingInfected}}\sim N\left[ 0.3,{0.03}^{2} \right]$$

and $P_{\mathrm{BeingInfected}}$ of an agent with 100 CumuResource is

$$P_{\mathrm{BeingInfected}}\sim N\left[ 0.1,{0.01}^{2} \right]$$

when $I\mathrm{nequality}=10$, $P_{\mathrm{BeingInfected}}$ of an agent with 0 CumuResource is

$$P_{\mathrm{BeingInfected}}\sim N\left[ 0.25,{0.025}^{2} \right]$$

and $P_{\mathrm{BeingInfected}}$ of an agent with 100 CumuResource is

$$P_{\mathrm{BeingInfected}}\sim N\left[ 0.15,{0.015}^{2} \right]$$

$P_{\mathrm{BeingInfected}}$in our simulation refers to the practice of Cuevas,^1^ which sets the probability of being infected in the range of 0.1 to 0.3. After being judged to be infected, a green agent will become a yellow agent, representing an infected state, and enter the incubation period.

**Rule 4: Falling ill**

Khalili et al.^2^ concluded that the average case fatality rate of COVID-19 patients was 0.02 (95% CI: 0.02, 0.03), the average time it took for patients to recover was 18.55 days (95% CI: 13.69, 23.41), and the average time to death was 15.93 days (95% CI: 13.07, 18.79). Rule 4 considers real-world statistics and employs them in the simulation.

**Rule 6: Resource allocation**

The goal of resource allocation is to help the accumulated resources of most agents approach the maximum value (100). Considering that the mean of the normal distribution of the initial resources is 30, the total amount of resources is set to 63000 ($T\mathrm{otal}R\mathrm{esource}=9000\times(100-30)$). Resources are allocated according to two principles. The first is the principle of demand. Yellow agents are infected with the virus but have not been detected, and their demand for resources should be the same as that of green agents. Blue agents still need resources for protection, so they should have the same weight as green agents. The weights of these three agents participating in resource allocation are classified as the first category, labeled $r_{1}=1$. Because of the need for treatment, red and orange agents require many resources; thus, their weight is classified as the second category ($r_{2}=1.5$). For the need to undergo treatment such as isolation, the third category ($r_{3}=1.25$) describes the weight of the purple agents, who have been detected to be infected, and its value ranges between those of the previous two categories. The second principle is the principle of inequality. Agents with more initial resources will be allocated more resources each day, and the weight of resources allocated by each agent is the proportion of its initial resources among the total initial resources of all agents. $R\mathrm{esource}P\mathrm{er}I\mathrm{nitial}$ will be recalculated every day until the last day of resource allocation. If the simulation fails to stop at this time, it will continue to operate without resource allocation. Once the agent’s accumulated resources reach 100, the agent no longer participates in the resource allocation for the remaining days.


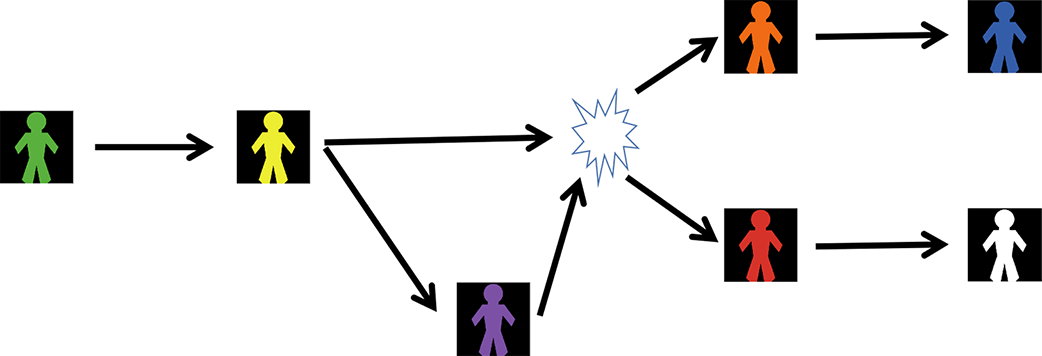


**Figure S5. A schematic diagram of the transformation of the agent in the simulation, with different colors representing different disease states.**

Green agents represent healthy individuals; yellow agents represent those in the incubation period; purple agents represent those who have tested positive; red agents represent those in the disease stage, with an ultimate fate of death; and white agents represent those who have died. Individuals who pass the incubation period turn orange, and blue agents represent those who have recovered.


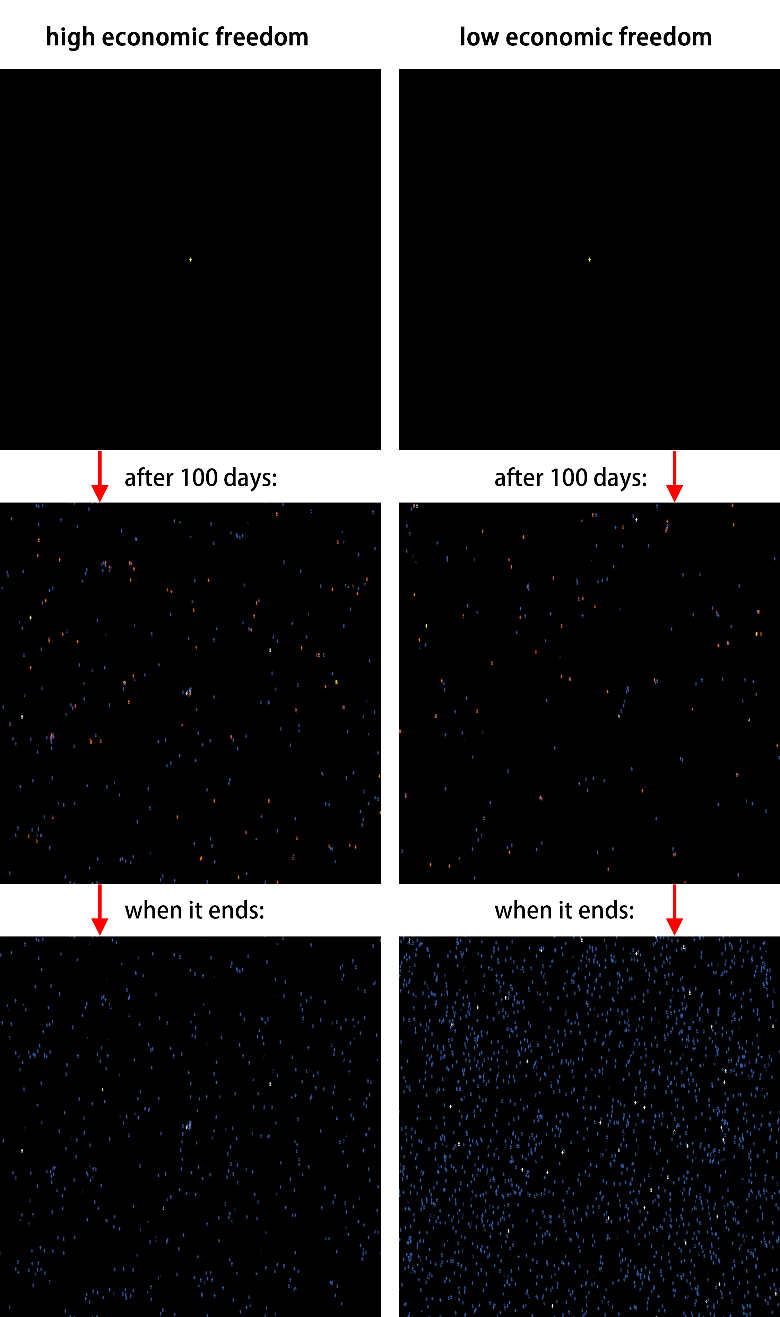


**Figure S6. Evolution process of the evolutionary game model.**

To clearly show the changes in the number of infected agents, green agents were changed to black. Yellow and purple agents were in the incubation period. Orange and red agents were in the disease-onset period. Blue represents recovery, and white represents death.

**
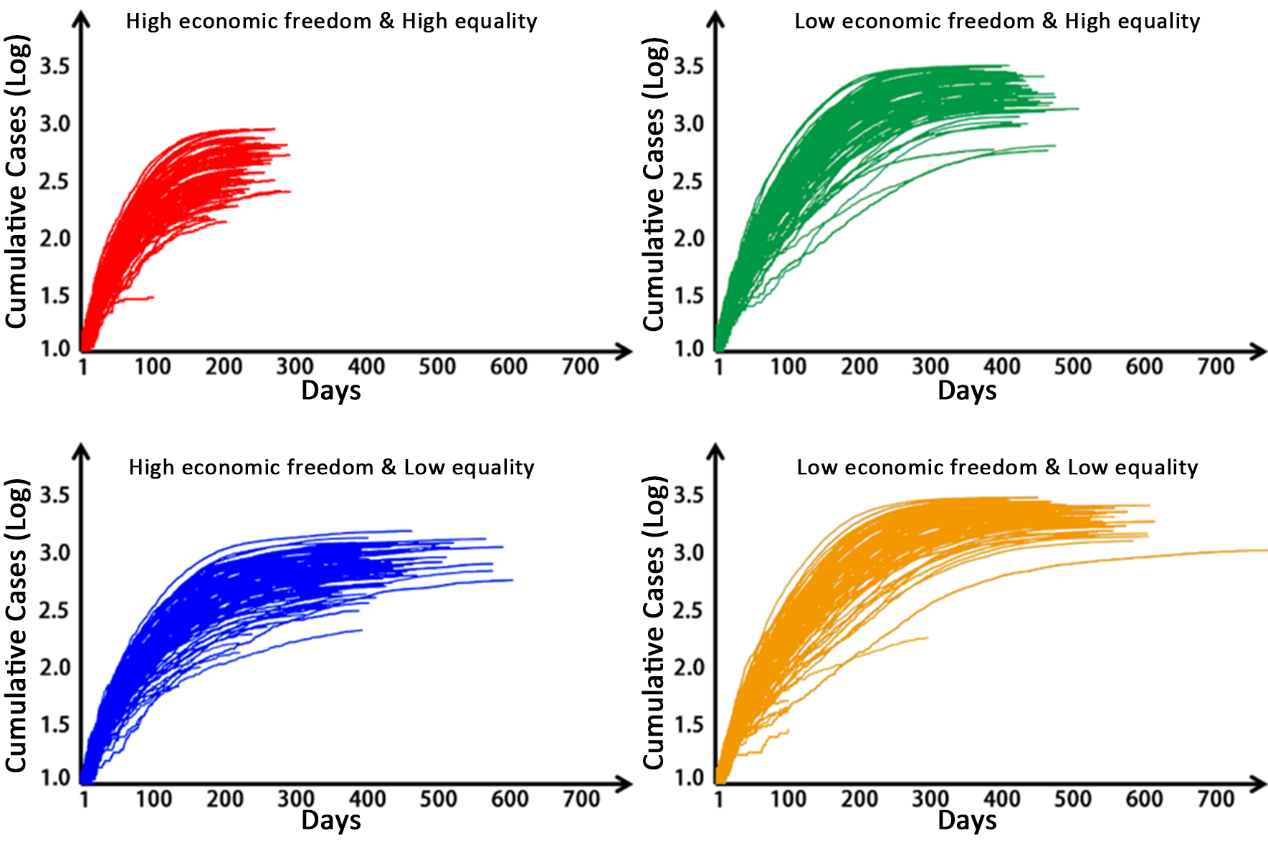
**

**Figure S7. The evolution of case numbers in four scenarios.**

**Discussion**

Many researchers have used agent-based modeling to study COVID-19-related issues. Cuevas studied the risk of people contracting COVID-19 in various settings.^2^ Gharakhanlou & Hooshangi explored the spatiotemporal laws of the spread of COVID-19 in a city in Iran.^3^ Similar to this study, Gelfand et al. used the evolutionary game model to verify that tight groups cooperate faster than loose groups under threats such as COVID-19.^4^ The purpose of the evolutionary game model used in this study was to determine the causal relationship between economic freedom and the speed of pandemic control; i.e., changes in economic freedom would be met with corresponding changes in the speed of pandemic control. Consistent with previous studies,^5–7^ we have proven that agent-based modeling is a powerful tool for studying the evolution of infectious diseases. We posit that the role of economic freedom was reflected in the speed of resource allocation by each society because an environment with high economic freedom could allocate a large amount of available resources in a short period of time. The results showed that the model replicated the effect of economic freedom and equality on the speed of pandemic control; specifically, economic freedom could positively affect pandemic control, and equality moderated the effect. However, this result provided evidence for the conclusion that economic freedom affects pandemic control, which is summarized in public data. It also indicated that resource allocation is the mechanism by which economic freedom affects pandemic control. In addition, the model verified the moderating effect of equality. Equality was manipulated as the gap between the rich and poor in terms of initial resources, and the initial resources determined the weight of resource distribution. We observed that despite the slight differences from the results based on public data, equality moderates the positive impact of economic freedom on the speed of pandemic control. When the pandemic struck, people who were economically disadvantaged were the first to experience the consequences, as the widespread structural inequality in society restricted this part of the population's access to appropriate resources.^8^ Infectious diseases continue to spread because not all members have the ability to protect themselves. This explains why economic freedom did not play a positive role in pandemic control in the absence of equality.

**Study 3a**

**Methods**

**Priming materials**

The information read by the participants in the economic freedom group was the following: “Imagine you live in an economically free society. Companies can easily obtain business licenses and do not need to spend much time on administrative procedures. Companies can produce what they think is the most profitable product while ignoring those that society most needs but that are not profitable. Companies can require employees to work long hours and terminate a contract when the employee is no longer needed. During production, companies produce as many commodities as they wish, regardless of whether the market has the capacity to consume them. Individually, you can choose the work you most want. You are not protected by a minimum wage; the harder you work, the more you get. Since the tax is low, you enjoy almost all of your income. The currency circulation in the market remains stable; thus, you can predict your future income and expenditures, and it is quite easy to make investment plans.”

The information read by the participants in the economic unfree group was the following: “Imagine you live in a society that is not economically free. Companies need to undergo careful inspection and review during the process of applying for business licenses. Companies need to decide what products to produce according to relevant regulations. Companies cannot require employees to work long hours, nor can they terminate contracts with employees. During production, companies need to determine the production and sales quantities of goods according to macroeconomic policy to reduce unnecessary waste of resources. Individually, you do not need to worry about your work because it is already arranged. You are protected by a minimum wage, and you can get a basic wage without too much effort. Your take-home pay will be decreased as part of the money goes to pay taxes. The currency circulation in the market is unstable, and you cannot predict your future income or expenditures and cannot make investment plans.”

The information read by the participants in the neutral group was the following: “Imagine you live in an ordinary society: The basic components of society, such as enterprises, schools, and hospitals, are all operating normally. Everyone in society leads an ordinary life. There is nothing in the news that worries you and nothing that makes you happy. After receiving your education in an ordinary school, you have found an ordinary but busy job. You want to improve the quality of life for yourself and your family, so you have been working hard, hoping to obtain a promotion. You have your favorite sports activities and spend time with your friends after work.”

**Control variables:**

**Self-constructs.** The self-constructs scale compiled by Singelis contains 24 items.^9^ Twelve items were used to measure the participants' interdependent self-constructs, and 12 items were used to measure their independent self-constructs. A 7-point Likert scale was used, with “strongly disagree” at 1 and “strongly agree” at 7. The higher the score is, the more participants make evaluations based on their relationship with others and their position in the group.

**General distrust.** The general distrust scale compiled by Yamagishi was used to measure the general distrust of participants.^10^ A five-item scale was used to measure the individual’s distrust of others. Items 1 and 4 captured the mentality that “others are basically honest”, and items 2, 3, and 5 captured the mentality that “trusting other people is Risky”. A 7-point Likert scale was used, with “strongly disagree” at 1 and “strongly agree” at 7.

**Tightness.** The tightness scale compiled by Gelfand et al. was used to measure tightness.^11^ We used a short version of the scale with 6 items. The scale measured the participants’ perceptions of the norms and standards of society. For example, “There are many social norms that people are supposed to abide by in this country.” A 7-point Likert scale was used, with “strongly disagree” at 1 and “strongly agree” at 7. The higher the score of the participants is, the higher their perception of social norms.

**Just world.** The just world scale compiled by Dalbert was used.^12^ The scale contains 7 items, for example, “I am usually treated fairly”. A 6-point Likert scale was used, with “strongly disagree” at 1 and “strongly agree” at 6. A higher score represented a higher perception of justice.

**Subjective socioeconomic status (SES).** Using a single-item measurement developed by Adler, Epel, Castellazzo, & Ickovics, the participants evaluated their socioeconomic status on a 10-level scale.^13^ The bottom of the scale represented the lowest socioeconomic status (coded as 1), and the top represented the highest socioeconomic status (coded as 10). The higher the number is, the higher the participant's SES.

**Social value orientation (SVO).** The Social Value Orientation scale compiled by Van Lange, De Bruin, Otten, & Joireman was used.^14^ The scale consists of 9 questions, each of which describes a decision-making situation with 3 options. The three options represent “competition”, “individual”, and “cooperation”. According to the number of options associated with the 9 decisions, participants are assigned to a type. Specifically, those who chose no less than 6 responses of “competition” were competitive, those who chose no less than 6 responses of “individual” were individualistic, those who chose no less than 6 responses of “cooperation” were prosocial, and the remaining were unidentified.

**Study 3b**

**Methods**

**Priming materials**

The information read by the participants in the economic freedom-equality group was the following: “Imagine you live in an economically free society: Companies can easily obtain business licenses and do not need to spend much time on administrative procedures. Companies can produce what they think is the most profitable product while ignoring those that society most needs but that are not profitable. Companies can require employees to work long hours and terminate contracts when they are no longer needed. During production, companies produce as many commodities as they wish, regardless of whether the market has the capacity to consume them. Regardless of ethnicity, nationality and religion, companies will treat you equally. Regardless of your age, sexual orientation and gender, companies will give everyone the same job opportunity, and you can choose your job freely. Because companies are going to pay you as little as possible, people who are very low paid can obtain financial subsidies from the labor union. You will receive social support from the labor union when you are unable to work due to illness or accidents. Since the tax is low, you enjoy almost all the income. The currency circulation in the market remains stable; thus, you can predict your future income and expenditure, and it is quite easy to make investment plans.”

The information read by the participants in the economic freedom-inequality group was the following: “Imagine you live in an economically free society: Companies can easily obtain business licenses and do not need to spend much time on administrative procedures. Companies can produce what they think is the most profitable product while ignoring those that society most needs but that are not profitable. Companies can require employees to work long hours and terminate contracts when they are no longer needed. During production, companies can produce as many commodities as they wish, regardless of whether the market has the capacity to consume them. Individually, you can choose the work you most want. However, companies have certain recruitment preferences, such as hiring younger candidates. Companies will exploit you and pay you as little as possible. In addition, you are not protected by the minimum wage. Since the tax is low, you enjoy almost all the income. The currency circulation in the market remains stable; thus, you can predict your future income and expenditure, and it is quite easy to make investment plans. Those with more wealth are more likely to acquire more money. People with more wealth can use their advantaged position to acquire political power, so they can not only maintain their vested interests but also pursue more interests. For example, the rich can set a high bar for education admittance to block the poor out.”

The material read by the participants in the economic unfree-equality group was the following: “Imagine you live in an economically unfree society: Companies need to undergo careful inspection and review during the process of applying for business licenses. Companies need to decide what products to produce according to relevant regulations. Companies cannot require employees to work long hours, nor can they terminate contracts with employees. During production, companies need to determine the production and sales quantities of goods according to macroeconomic policy to reduce the unnecessary waste of resources. Regardless of race, nationality and religion, companies will treat you equally. Regardless of your age, sexual orientation and gender, companies will give everyone the same job opportunity, and you can choose your job freely. Individually, you do not need to worry about your work because it is already arranged. You are protected by the minimum wage, and you can earn a basic wage without too much effort. You will receive social support from the labor union when you are unable to work due to illness or accidents. The earnings from your work will be deducted to pay taxes. The more you are paid, the more taxes you have to pay. The currency circulation in the market is unstable, and you cannot predict your future income or expenditure or make investment plans.”

The material read by the participants in the economic unfree-inequality group was the following: “Imagine you live in an economically unfree society: Companies need to undergo careful inspection and review during the process of applying for business licenses. Companies need to decide what products to produce according to relevant regulations. Companies cannot require employees to work long hours, nor can they terminate contracts with employees. During production, companies need to determine the production and sales quantities of goods according to macroeconomic policy to reduce the unnecessary waste of resources. Individually, you do not need to worry about your work because it is already arranged. However, companies have certain preferences, such as assigning good jobs to younger people first. You are protected by the minimum wage, and you can earn a basic wage without too much effort. However, companies will exploit you and pay you as little as possible. The earnings from your work will be deducted to pay taxes. However, the tax rate is fixed, regardless of how much you are paid. The currency circulation in the market is unstable, and you cannot predict your future income or expenditure and cannot make investment plans. People with more wealth can use their advantaged position to acquire political power, so they can not only maintain their vested interests but also pursue more interests. For example, the rich can set a high bar for education admittance to block the poor out.”

**Control variables:**

Same as study 3a.

**Discussion**

The goal of Study 3 was to explore how economic freedom affects resource allocation at the individual level. In study 3a, we discussed the influence of economic freedom on individual behavior. In study 3b, we added the equality variable to further explore the moderating role of equality in economic freedom and individual behavior. The participants in the economic freedom group perceived higher economic freedom than those in the economic unfree group, which indicated that the economic freedom priming was successful. The participants in the equal group perceived higher equality than those in the unequal group, which indicated that the priming of equality was successful. The three resource tasks in the context of COVID-19 were designed according to cooperation, risk aversion, and loss aversion. The resources generated by the participants in the tasks were considered to be led by the corresponding psychological mechanisms. Briefly, this study explores the possibility that economic freedom may influence individuals' cooperative behavior and, thus, further influence the speed of social resource allocation. A previous study demonstrated that economic freedom promotes trust.^15^ Therefore, we suspect that economic freedom may promote individuals' trust in others, which makes individuals more willing to cooperate with others. Further research showed that in an equal society, high economic freedom endows individuals with a greater tendency to cooperate. It is possible that individuals in highly economically free societies are more likely to trust others and trust the government to distribute resources fairly. However, in an unequal society, the government does not allocate resources fairly. Therefore, economic freedom may not promote trust, leading to the failure of economic freedom to affect the cooperative behavior of individuals. In a society with high equality, individuals in a society with high economic freedom are more willing to cooperate and make contributions to the collective, thus making more resources available in a society with high economic freedom. More available resources can be used to better protect individuals from infection. Even if individuals become infected, they can be isolated and treated in a timely manner to prevent the infection from spreading, which ultimately promotes faster pandemic control in societies with economic freedom.

**References**

1 Haan JD, Sturm JE. On the relationship between economic freedom and economic growth. *European Journal of Political Economy* 2000; **16**, 215-41.

2 Cuevas E. An agent-based model to evaluate the COVID-19 transmission risks in facilities. *Computers in Biology and Medicine* 2020; **121:** 103827.

3 Gharakhanlou NM, Hooshangi N. Spatio-temporal simulation of the novel coronavirus (COVID-19) outbreak using the agent-based modeling approach (case study: Urmia, Iran). *Informatics in Medicine Unlocked* 2020; **20:** 100403.

4 Gelfand MJ, Jackson JC, Pan X, et al. The relationship between cultural tightness–looseness and COVID-19 cases and deaths: A global analysis. *The Lancet Planetary Health* 2021; **5:** e135–e44.

5 Epstein JM, Cummings DAT, Chakravarty S, Singha RM, Burke DS. *Toward a containment strategy for smallpox bioterror: An individual-based computational approach*. Brookings Institution Press 2004.

6 Gharakhanlou NM, Mesgari MS, Hooshangi N. Developing an agent-based model for simulating the dynamic spread of Plasmodium vivax malaria: A case study of Sarbaz, Iran. *Ecological Informatics* 2019; **54:** 101006.

7 Rao DM, Chernyakhovsky A, Rao V. Modeling and analysis of global epidemiology of avian influenza. *Environmental Modelling & Software* 2009; **24:** 124–34.

8 Lynch JW, Smith GD, Kaplan GA, House JS. Income inequality and mortality: Importance to health of individual income, psychosocial environment, or material conditions. *BMJ* 2000; **320:** 1200–04.

9 Singelis TM. The measurement of independent and interdependent self-construals. *Personality and Social Psychology Bulletin* 1994; **20:** 580–91.

10 Yamagishi T. The provision of a sanctioning system as a public good. *Journal of Personality and Social Psychology* 1986; **51:** 110–16.

11 Gelfand MJ, Raver JL, Nishii L, et al. Differences between tight and loose cultures: A 33-nation study. *Science* 2011; **332:** 1100–04.

12 Dalbert C. The world is more just for me than generally: About the personal belief in a just world scale’s validity. *Social Justice Research* 1999; **12:** 79–98.

13 Adler NE, Epel ES, Castellazzo G, Ickovics JR. Relationship of subjective and objective social status with psychological and physiological functioning: Preliminary data in healthy white women. *Health Psychology* 2000; **19:** 586–92.

14 Van Lange PAM, De Bruin EMN, Otten W, Joireman JA. Development of prosocial, individualistic, and competitive orientations: Theory and preliminary evidence. *Journal of Personality and Social Psychology* 1997; **73:** 733–46.

15 Berggren N, Jordahl H. Free to trust: economic freedom and social capital. Kyklos 2010; 59(2): 141-69.
